# Protocatechuic acid attenuates isoproterenol-induced cardiac hypertrophy via downregulation of ROCK1–Sp1–PKCγ axis

**DOI:** 10.1038/s41598-021-96761-2

**Published:** 2021-08-30

**Authors:** Liyan Bai, Hae Jin Kee, Xiongyi Han, Tingwei Zhao, Seung-Jung Kee, Myung Ho Jeong

**Affiliations:** 1 Heart Research Center, Chonnam National University Hospital, 42 Jebong-ro, Dong-gu, Gwangju, 61469 Republic of Korea; 2 Hypertension Heart Failure Research Center, Chonnam National University Hospital, Gwangju, 61469 Republic of Korea; 3 Department of Laboratory Medicine, Chonnam National University, Medical School and Hospital, Gwangju, 61469 Republic of Korea; 4 Department of Cardiology, Chonnam National University Medical School, Gwangju, 61469 Republic of Korea

**Keywords:** Molecular biology, Cardiology, Molecular medicine

## Abstract

Cardiac hypertrophy is an adaptive response of the myocardium to pressure overload or adrenergic agonists. Here, we investigated the protective effects and the regulatory mechanism of protocatechuic acid, a phenolic compound, using a mouse model of isoproterenol-induced cardiac hypertrophy. Our results demonstrated that protocatechuic acid treatment significantly downregulated the expression of cardiac hypertrophic markers (*Nppa, Nppb*, and *Myh7*), cardiomyocyte size, heart weight to body weight ratio, cross-sectional area, and thickness of left ventricular septum and posterior wall. This treatment also reduced the expression of isoproterenol-induced ROCK1, Sp1, and PKCγ both in vivo and in vitro. To investigate the mechanism, we performed knockdown and overexpression experiments. The knockdown of ROCK1, Sp1, or PKCγ decreased the isoproterenol-induced cell area and the expression of hypertrophic markers, while the overexpression of Sp1 or PKCγ increased the levels of hypertrophic markers. Protocatechuic acid treatment reversed these effects. Interestingly, the overexpression of Sp1 increased cell area and induced PKCγ expression. Furthermore, experiments using transcription inhibitor actinomycin D showed that ROCK1 and Sp1 suppression by protocatechuic acid was not regulated at the transcriptional level. Our results indicate that protocatechuic acid acts via the ROCK1/Sp1/PKCγ axis and therefore has promising therapeutic potential as a treatment for cardiac hypertrophy.

## Introduction

Cardiac hypertrophy is characterized by several parameters, such as increased heart size, upregulated protein synthesis, induction of fetal gene program, and aberrant organization of the sarcomere structure^1^. Sustained cardiac hypertrophy is not a compensatory state and can affect heart muscle remodeling and fibrosis, often progressing to heart failure^2^. In response to pathological insults, intracellular signal-transduction pathways have been shown to control the growth of the adult myocardium. Mitogen-activated protein kinase, G-protein coupled receptors, phosphoinositide 3-kinase, peroxisome proliferator-activated receptor, and small G proteins (RhoA, Rac, and Cdc42) are key mediators involved in the regulation of cardiac hypertrophy^3–7^. Rho-associated coiled-coil containing kinases (ROCKs) are the downstream targets of the small GTP-binding protein RhoA. ROCKs have two isoforms: ROCK1 and ROCK2; however, both isoforms play different roles in cardiac hypertrophy^8^. Therefore, the connection between ROCKs and cardiac hypertrophy needs to be investigated.

Protein kinase C (PKC) isoforms have also been shown to play an important role in cardiac contraction, hypertrophy, and signal transduction pathways^9–11^. Among classical PKC isoforms, PKCα is implicated in the stimulation of cardiac hypertrophy and the regulation of contractility^12–14^, while studies investigating the involvement of PKCβ in cardiac hypertrophy show conflicting results^15,16^. PKCδ and PKCε, members of the novel PKC subfamily, also play a role in cardiac hypertrophy^17,18^; however, the function of PKCγ in the heart remains unknown.

Atrial natriuretic peptide (ANP) and brain natriuretic peptide (BNP) are well-known markers of cardiac hypertrophy. ANP and BNP are regulated by several transcription factors. For example, transcription factors GATA binding factor 4 (GATA4) and GATA6 bind to the GATA motif located in the promoters of ANP and BNP, leading to the expressions of fetal genes^19,20^. It has been reported that GATA4 and GATA6 are involved in the induction of cardiac hypertrophy in vivo and *in vitro*^21,22^. Another transcription factor, located upstream of GATA4, is specificity protein 1 (Sp1); it upregulates the expression of GATA4 and ANP in cardiomyoblast cells^23^. Sp1 is also involved in cell growth, differentiation, and cancer^24,25^.

Protocatechuic acid (3,4-dihydroxybenzoic acid) is found in vegetable, fruits, and rice, including in chicory, plums, grapes, and grain brown rice. It has been shown to improve vasodilation in apolipoprotein E-deficient mice with atherosclerosis via the eNOS-mediated endothelium-dependent mechanism^26^. Protocatechuic acid has also been reported to have anti-osteoarthritic, anti-asthmatic, anti-cancer, anti-hyperglycemic, antioxidant, and anti-inflammatory effects ^27–29^. Protocatechuic acid has structural similarity with gentisic acid (2,5-dihydroxybenzoic acid). Hydroxybenzoic acid isomers have been found in many plants and have been regarded as protective mediators in cardiovascular system^30^. We previously reported that gentisic acid attenuated cardiac hypertrophy through the inhibition of the ERK1/2 MAPK signaling pathway^31^. Based on these results, we hypothesized that protocatechuic acid could also reverse the pathogenesis of this disease.

In this study, we investigated the effect of protocatechuic acid on cardiac hypertrophy both in vitro and in vivo, as well as the molecular mechanisms involved in these processes. Here, we showed that transcription factor Sp1 induced cardiomyocyte hypertrophy, while protocatechuic acid reversed these effects by inhibiting the ROCK1-Sp1-PKCγ pathway.

## Results

### Protocatechuic acid reduced isoproterenol-induced cardiomyocyte hypertrophy in H9c2 cells

**Figure 1:**
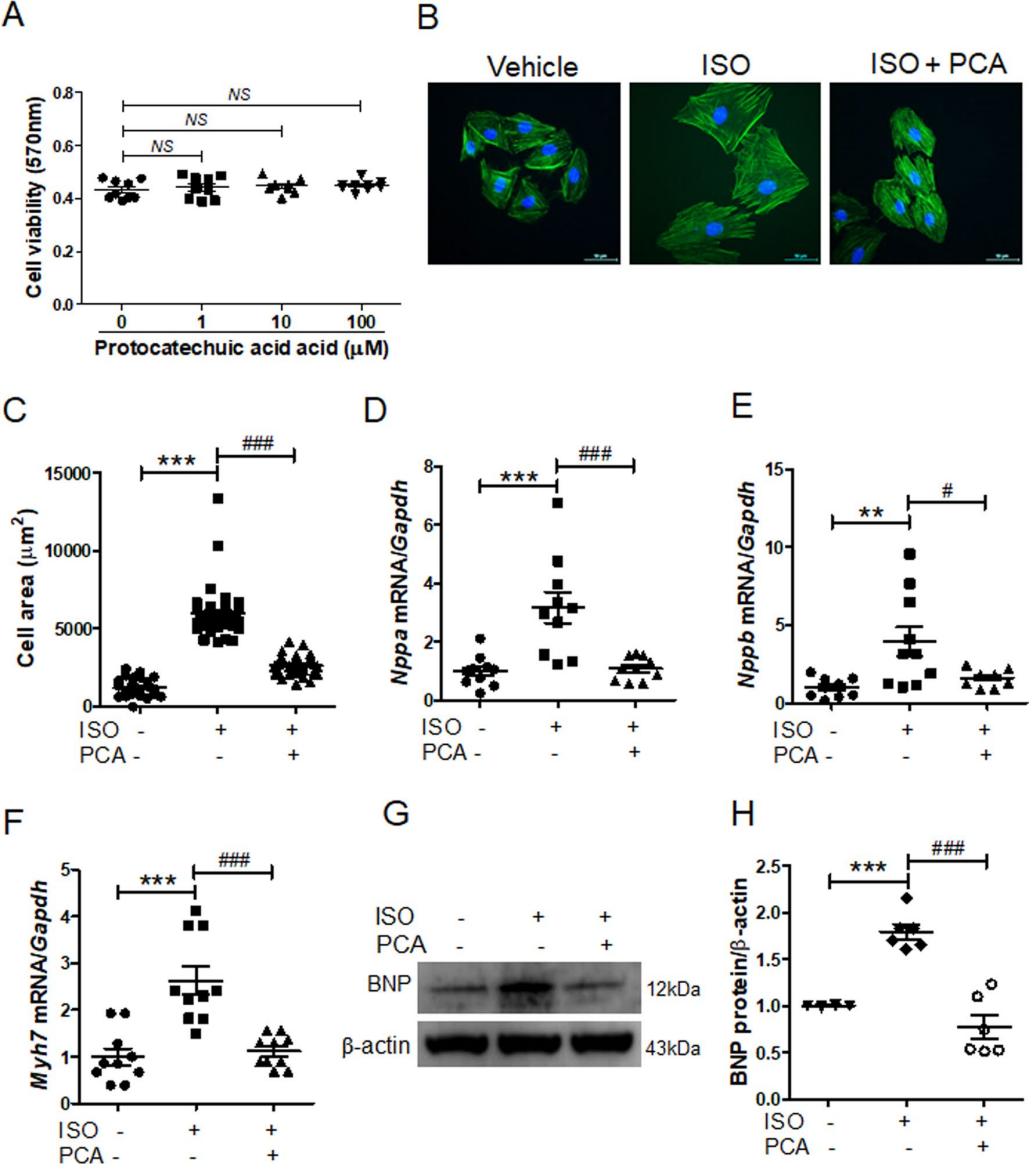
Protocatechuic acid reduces isoproterenol-induced cardiomyocyte hypertrophy in vitro. **(A)** H9c2 cells were treated with protocatechuic acid (0, 1, 10, 100 μM) for 24 h as indicated and cell viability was determined using the MTT assay (n = 8). NS indicates not significant. (**B**,**C**) H9c2 cells were serum-starved overnight and then treated with vehicle or protocatechuic acid in the presence or absence of isoproterenol (10 μM) for 24 h. Cell size was quantified by measuring the cell surface area of Alexa Fluor 488 phalloidin-stained cells (n = 40 cells). Scale bar = 50 μm. ***P < 0.001; ^*###*^*P* < 0.001. (**D‒H**) H9c2 cells were treated with isoproterenol (10 μM) in the presence or absence of protocatechuic acid (10 μM) for 6 h. The mRNA expression levels of *Nppa* (**D**)*, Nppb* (**E**), and *Myh7* (**F**) were determined by RT-PCR and normalized to *Gapdh* (n = 10). **P < 0.01 and ***P < 0.001; ^*#*^*P* < 0.05 and ^*###*^*P* < 0.001. (**G**) Representative western blot images are shown; β-actin was used as a loading control. (**H**) Quantification of BNP protein levels (n = 6). ***P < 0.001; ^*###*^*P* < 0.001. ISO, isoproterenol; PCA, protocatechuic acid. Data are presented as the mean ± S.E. Statistical analysis: one-way ANOVA followed by Bonferroni post hoc tests. Graphs were prepared using GraphPad 5.0.

Before we investigated the effect of protocatechuic acid on cardiac hypertrophy, we evaluated protocatechuic acid toxicity in H9c2 cells. MTT assay showed that protocatechuic acid (up to 100 μM concentration) did not affect cell viability (Fig. 1A). Next, we evaluated the effect of protocatechuic acid on isoproterenol-induced cell hypertrophy in vitro and observed that protocatechuic acid treatment significantly reduced cell size (Fig. 1B,C). Furthermore, protocatechuic acid decreased isoproterenol-induced mRNA levels of *Nppa, Nppb,* and *Myh7* (Fig. 1D‒F). Western blotting confirmed that protocatechuic acid reduced isoproterenol-induced BNP protein expression (Fig. 1G,H).

### Protocatechuic acid attenuated cardiac hypertrophy in isoproterenol-infused mice

**Figure 2:**
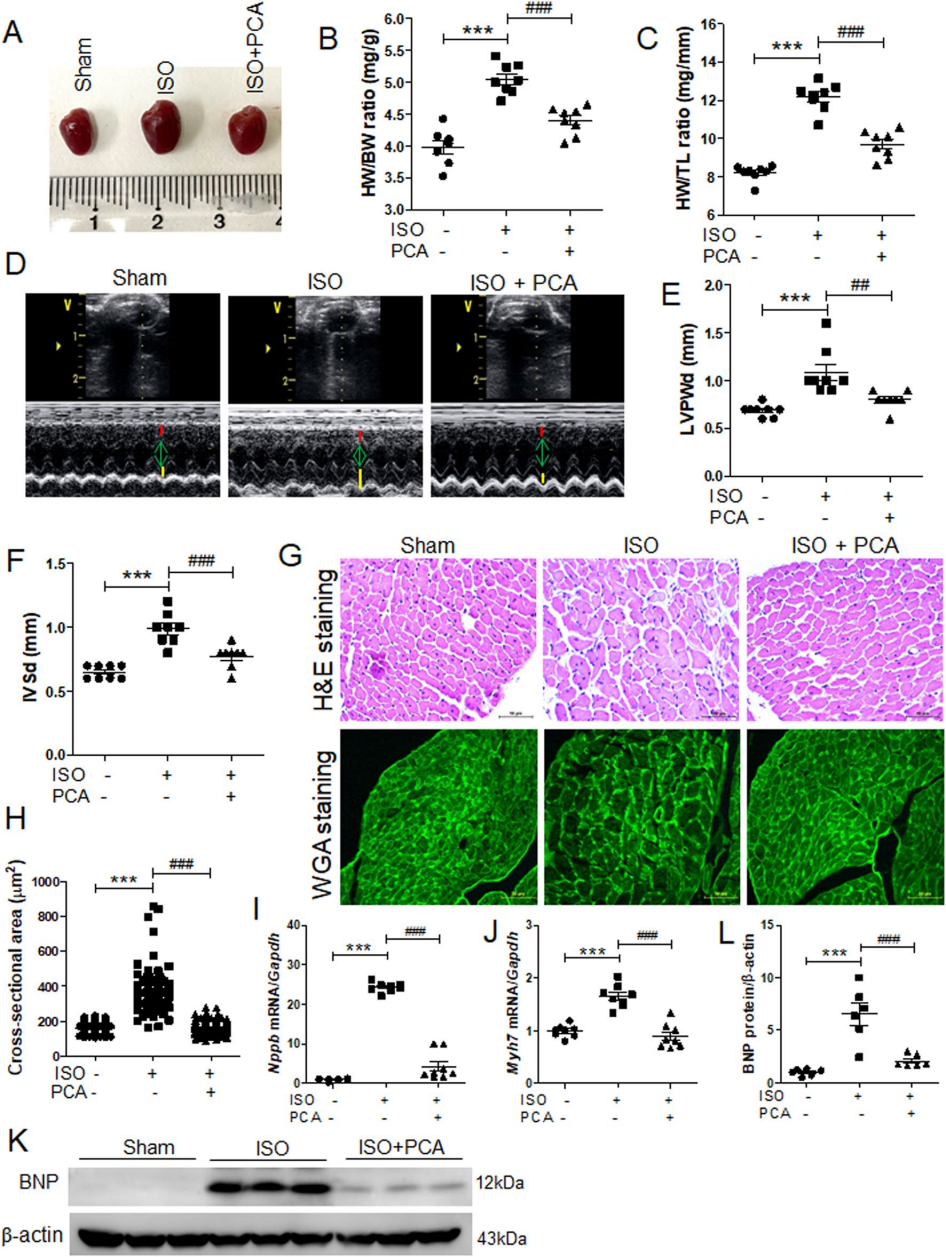
Protocatechuic acid attenuates isoproterenol-induced cardiac hypertrophy in vivo. (**A**) Representative images of gross hearts from sham, isoproterenol, and isoproterenol + protocatechuic acid treated mice. (**B**,**C**) Heart weight to body weight ratio (HW/BW) and heart weight to tibia length ratio (HW/TL) of the mice described in (**A**) (n = 8). ***P < 0.001; ^*###*^*P* < 0.001. (**D**) Representative B-mode and M-mode echocardiograms are shown. Cardiac hypertrophy was determined by echocardiography after 5 days of isoproterenol administration. (**E,F**) Left ventricular posterior wall thickness (LVPWd, mm) and interventricular septum thickness (IVSd, mm) in mice (n = 8). ***P < 0.001; ^*##*^*P* < 0.01 and ^*###*^*P* < 0.001. ISO, isoproterenol; PCA, protocatechuic acid. (**G**) Representative images of H&E (upper panel) and wheat germ agglutinin (WGA, lower panel) staining of left ventricle papillary muscle from sham, isoproterenol, and isoproterenol + protocatechuic acid-treated mice. Scale bar = 50 μm. (**H**) Cross-sectional area quantification of H&E-stained hearts described in (**G**), n = 161 cells. ***P < 0.001; ^*###*^*P* < 0.001. (**I**,**J**) Total RNA was isolated from the hearts of mice (n = 8). mRNA levels of *Nppb* (**I**) and *Myh7* (**J**) were determined by RT-PCR and normalized to *Gapdh*. ***P < 0.001; ^*###*^*P* < 0.001. (**K**,**L**) Representative Western blot images of BNP and quantification (n = 6); β-actin was used as a loading control. ***P < 0.001; ^*###*^*P* < 0.001*.* Data are presented as the mean ± S.E. Statistical analysis: one-way ANOVA followed by Bonferroni post hoc tests. Graphs were prepared using GraphPad 5.0.

To verify whether protocatechuic acid can inhibit cardiac hypertrophy in vivo, mice were treated with protocatechuic acid (100 mg/kg/day) for 5 days following the isoproterenol infusion via the osmotic minipump. This dose of protocatechuic acid was selected based on previously published studies^32^. As expected, isoproterenol infusion increased the gross heart size, heart weight to body weight ratio, and heart weight to tibia length ratio; however, these increases were significantly reduced by protocatechuic acid treatment (Fig. 2A‒C). To further confirm the anti-hypertrophic effects of protocatechuic acid, we performed echocardiography. Isoproterenol administration for 5 days increased the thickness of left ventricular posterior wall and interventricular septum; both parameters were significantly decreased by protocatechuic acid treatment (Fig. 2D‒F). Isoproterenol infusion did not affect the left ventricular end-diastolic dimension; however, it reduced the left ventricular end-systolic dimension (Supplementary Fig. 1A and B). These changes were reversed by protocatechuic acid treatment (Supplementary Fig. 1B). Furthermore, isoproterenol or protocatechuic acid did not affect the ejection fraction (EF) value (Supplementary Fig. 1C). To evaluate the effect of protocatechuic acid on cardiomyocyte morphology and cell size, heart tissues were stained with hematoxylin and eosin (H&E) and wheat germ agglutinin. The isoproterenol-induced increase of the cross-sectional area was reversed by protocatechuic acid treatment (Fig. 2G,H). Furthermore, the mRNA levels of *Nppb* and *Myh7* upregulated by isoproterenol infusion, were significantly decreased by protocatechuic acid treatment (Fig. 2I,J). BNP protein levels were also reduced in isoproterenol- and protocatechuic acid-treated mice (Fig. 2K,L), confirming the qRT-PCR results.

### Isoproterenol-induced cardiomyocyte hypertrophy was reversed by ROCK1 downregulation

**Figure 3:**
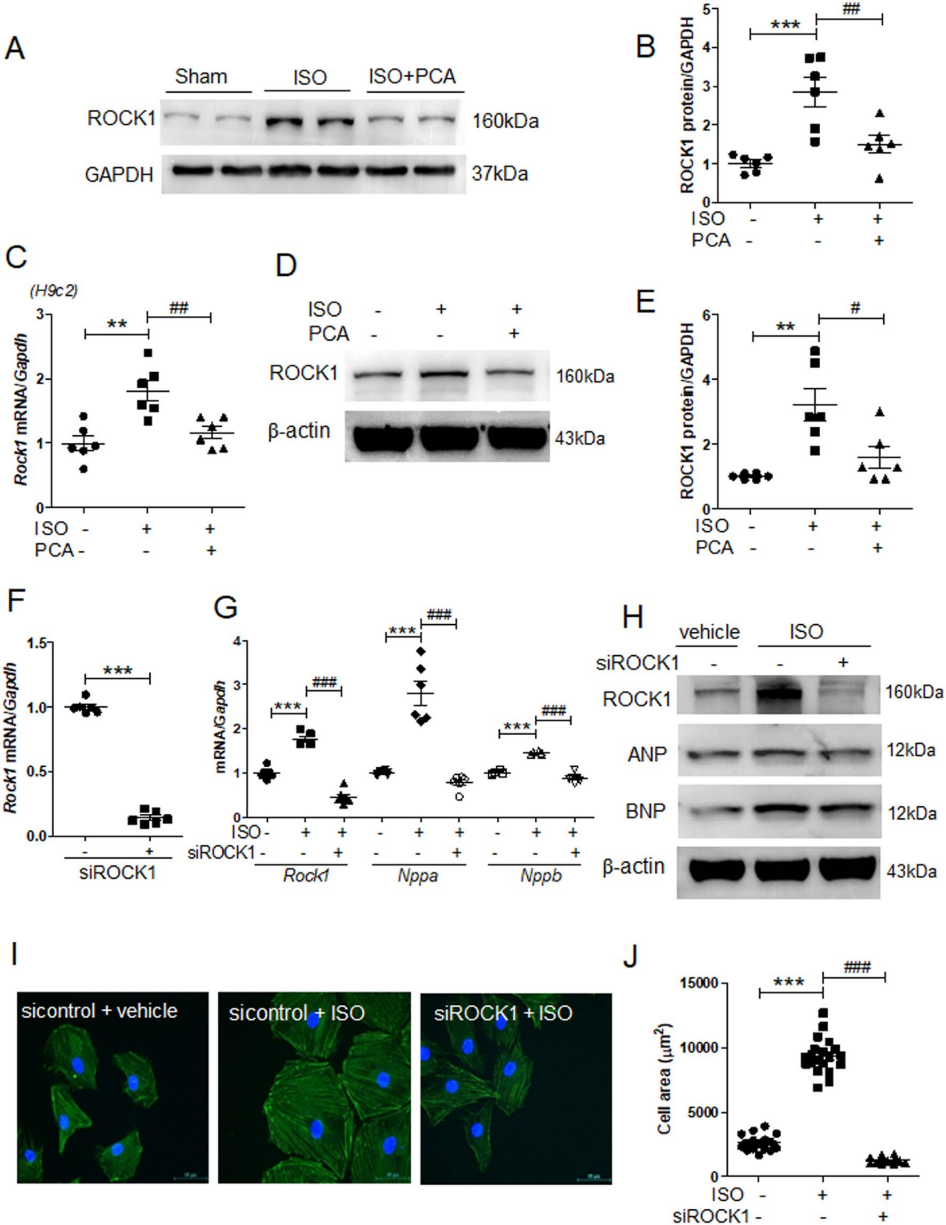
ROCK1 knockdown reduces isoproterenol-induced cardiomyocyte hypertrophy. (**A**) Heart tissues from sham, isoproterenol, and isoproterenol + protocatechuic acid treated mice were analyzed by western blotting using anti-ROCK1 and GAPDH antibodies. Representative images. (**B**) Quantification of ROCK1 protein levels in heart tissues (n = 6); the results were normalized to GAPDH. ***P < 0.001; ^*##*^*P* < 0.01. (**C − E**) H9c2 cells were serum starved overnight and then treated with isoproterenol (10 μM) in the presence or absence of protocatechuic acid (10 μM) for 6 h. (**C**) mRNA levels of *Rock1* (n = 6). **P < 0.01; ^*##*^*P* < 0.01. (**D**) Western blot analysis. Representative images of ROCK1. (**E**) Quantification of western blots presented in (**D,** n = 6). **P < 0.01; ^*#*^*P* < 0.05*.* (**F**) H9c2 cells were transfected with either control or ROCK1 siRNA for 2 days. The mRNA levels of *Rock1* were analyzed using RT-PCR. ***P < 0.001, Student’s *t*-test. (**G**) RT-PCR analysis of *Rock1, Nppa*, and *Nppb* expression in H9c2 cells transfected with either control or ROCK1 siRNA in the presence or absence of isoproterenol (10 μM, n = 6). ***P < 0.001; ^*###*^*P* < 0.001. (**H**) Western blot analysis of cells described in (**G**). Representative images of ROCK1, ANP, and BNP; β-actin was used as a loading control. (**I**) Representative merged images of H9c2 cells transfected with either control or ROCK1 siRNA, incubated with 10 μM isoproterenol for 24 h, and then stained. F-actin filaments (green) and DAPI (blue). Scale bar = 50 μm. (**J**) Cell area quantification of H9c2 cells cultured on 12-well plates (n = 20 cells). ***P < 0.001; ^*###*^*P* < 0.001. ISO, isoproterenol; PCA, protocatechuic acid. Data are presented as the mean ± S.E. Statistical analysis: one-way ANOVA followed by Bonferroni post hoc tests. Graphs were prepared using GraphPad 5.0.

To elucidate the mechanism of protocatechuic acid function, we decided to focus on ROCK1, the kinase involved in the regulation of cell shape in cardiac hypertrophy^4,33^. Protocatechuic acid treatment reduced ROCK1 protein expression levels in the heart tissues of isoproterenol-treated mice (Fig. 3A,B) and reversed the isoproterenol-induced *Rock1* mRNA and protein expression levels in H9c2 cells (Fig. 3C − E). To investigate the role of ROCK1 in cardiac hypertrophy, we transfected H9c2 cells with ROCK1 siRNA. As expected, ROCK1 siRNA reduced the levels of endogenous *Rock1* mRNA (Fig. 3F). Furthermore, both mRNA and protein expression levels of isoproterenol-induced *Rock1*, *Nppa* (ANP), and *Nppb* (BNP) were also significantly decreased by ROCK1 siRNA (Fig. 3G,H, Supplementary Fig. 2).

Next, we investigated the effect of ROCK1 downregulation on cardiomyocyte hypertrophy. In the absence of isoproterenol stimulation, there were no significant differences in cell size between control and ROCK1 siRNA groups (Supplementary Fig. 3); however, ROCK1 knockdown significantly decreased the isoproterenol-induced cell hypertrophy (Fig. 3I,J).

### Knockdown of Sp1 reduced the isoproterenol-induced cardiomyocyte size and the expression of hypertrophic markers

**Figure 4:**
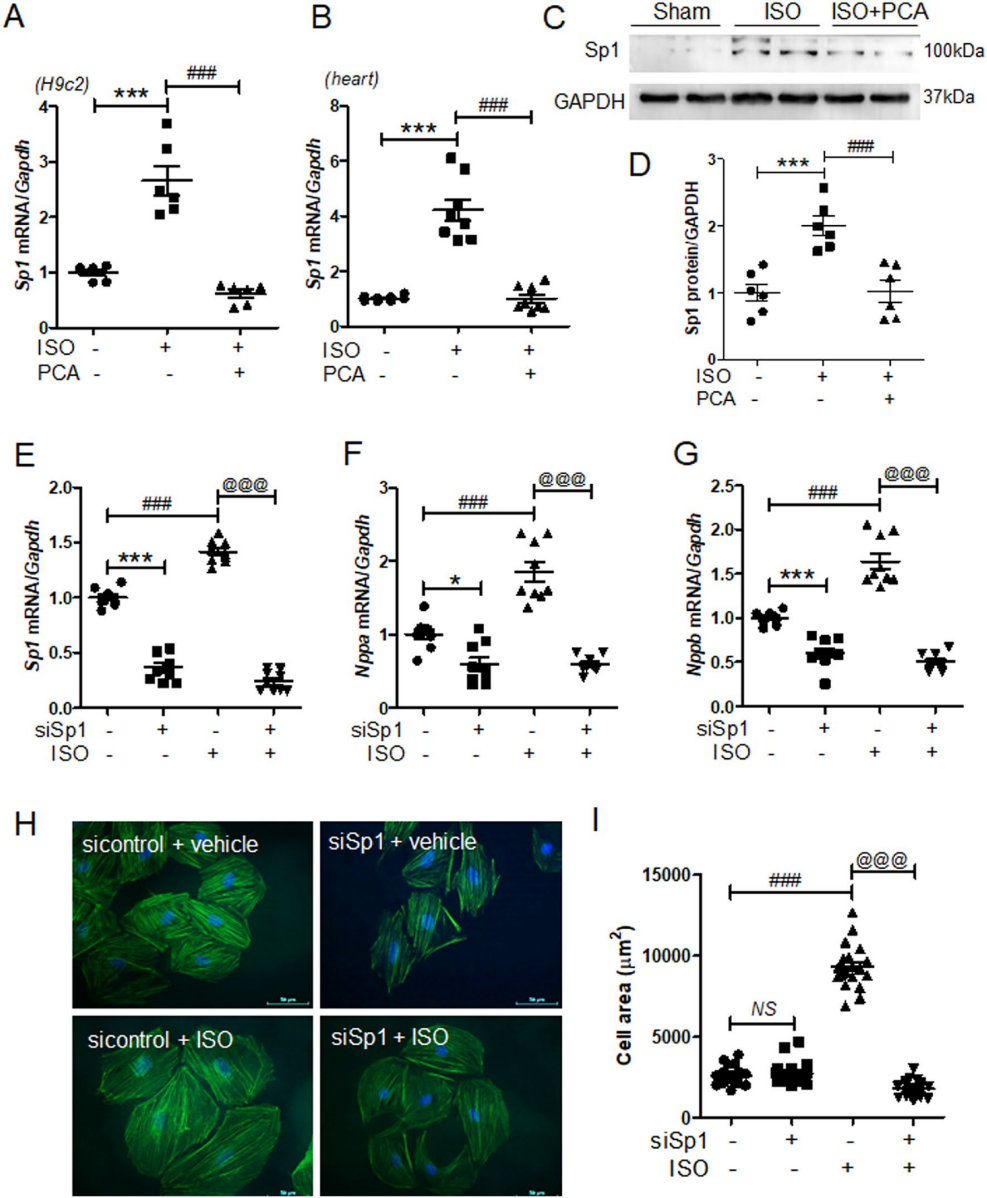
Sp1 knockdown reduces isoproterenol-induced cardiac hypertrophy in H9c2 cells. (**A**) *Sp1* mRNA levels in H9c2 cells treated with isoproterenol (10 μM) in the presence or absence of protocatechuic acid (10 μM, n = 6). ***P < 0.001; ^*###*^*P* < 0.001. (**B**) *Sp1* mRNA levels in heart tissues treated with protocatechuic acid (100 mg/kg/day) in the presence or absence of isoproterenol (25 mg/kg, n = 8). ***P < 0.001; ^*###*^*P* < 0.001. (**C**) Representative western blot images showing SP1 protein levels in mouse hearts. GAPDH was used as a loading control. (**D**) Quantification of SP1 protein levels (n = 6). ***P < 0.001; ^*###*^*P* < 0.001. (**E‒G**) H9c2 cells were transfected with control or Sp1 siRNA, serum starved overnight, and then treated with isoproterenol (10 μM). *P < 0.05 and ***P < 0.001; ^*###*^*P* < 0.001*; *^*@@@*^*P* < 0.001. mRNA levels of *Sp1*, *Nppa*, and *Nppb* (n = 9). (**H**) Merged images of H9c2 cells transfected with Sp1 siRNA, incubated with or without isoproterenol stimulation (10 μM) for 24 h, and then stained with Alexa Fluor 488 phalloidin (green) and DAPI (blue). (**I**) Cell size was quantified by measuring the cell surface area of the Alexa Fluor 488 phalloidin-stained cells (n = 20 cells). Scale bar = 50 μm. ^*###*^*P* < 0.001*; *^*@@@*^*P* < 0.001. Data are presented as the mean ± S.E. Statistical analysis: one-way ANOVA followed by Bonferroni post hoc tests. Graphs were prepared using GraphPad 5.0.

It has been reported that the expression of cardiac hypertrophy marker ANP is regulated by transcription factor Sp1^23,34^. Therefore, we evaluated the effect of protocatechuic acid treatment on Sp1 expression in vivo and in vitro. As expected, the isoproterenol stimulation increased the expression of *Sp1* mRNA; however, this effect was decreased by protocatechuic acid treatment both in H9c2 cells and in mouse heart tissues (Fig. 4A,B). Western blot analysis of mouse heart tissues showed similar results (Fig. 4C,D). To evaluate the effect of Sp1 knockdown on cardiac hypertrophy, H9c2 cells were transfected with Sp1 siRNA and then treated with isoproterenol. The knockdown of Sp1 successfully reduced the mRNA levels of *Sp1* (Fig. 4E), as well as *Nppa* and *Nppb*, in both control and isoproterenol-treated groups (Fig. 4F,G). In order to investigate whether the silencing of Sp1 affected the cell size, cells were stained with Alexa Fluor 488 phalloidin. In the absence of isoproterenol stimulation, *Sp1* knockdown did not have any effect on the cell size (Fig. 4H,I); however, in response to the stimulation, it completely reversed isoproterenol-induced cell hypertrophy (Fig. 4H,I).

### Protocatechuic acid reduced Sp1 overexpression-induced cardiac hypertrophy

**Figure 5:**
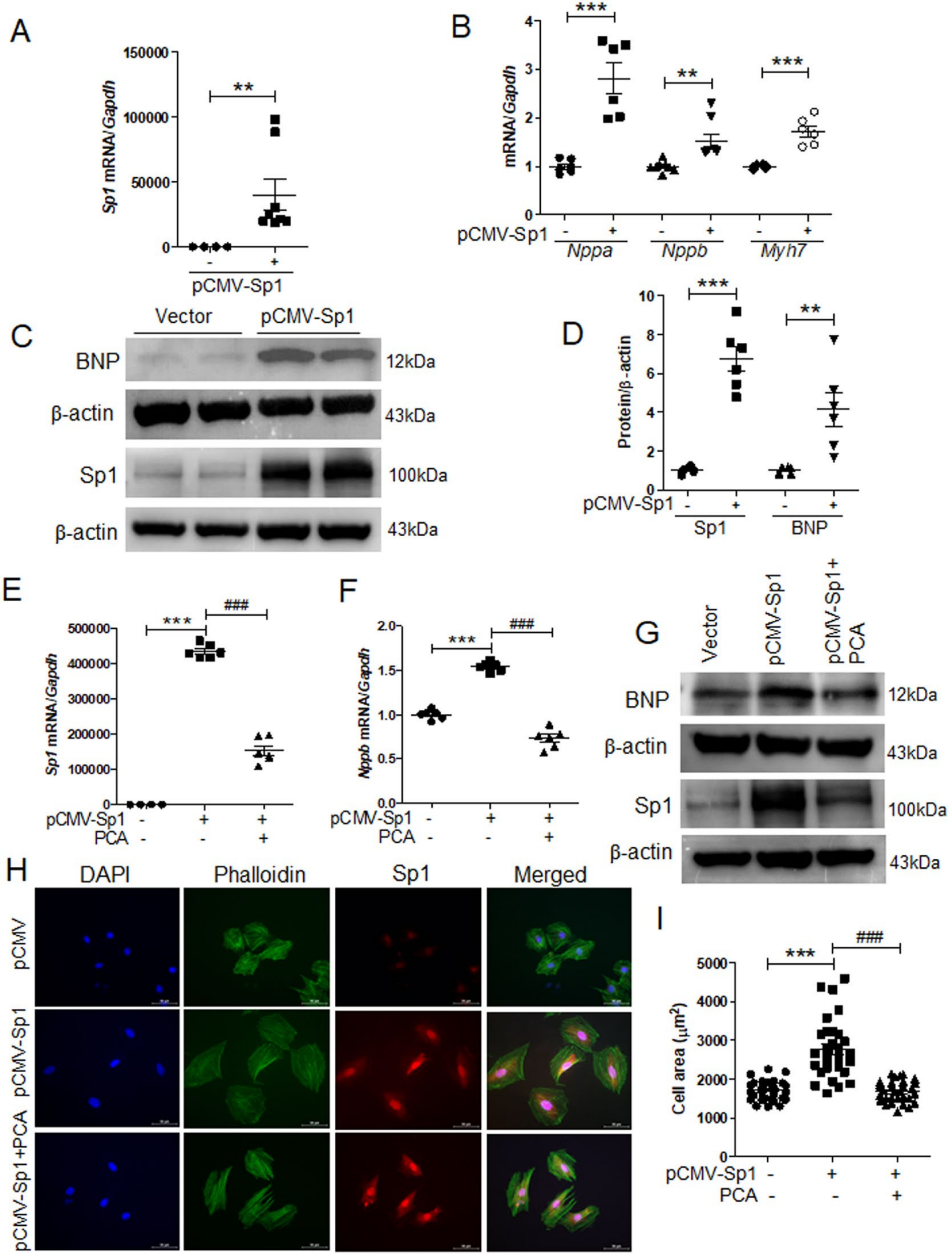
Protocatechuic acid ameliorates Sp1 overexpression-induced cardiac hypertrophy. H9c2 cells were transfected with empty vector or *pCMV-Sp1* for 24 h. (**A**) Transcript levels of mouse *Sp1* were quantified (n = 8). **P < 0.01, Student’s *t*-test. (**B**) The expression of hypertrophic marker genes (*Nppa* (n = 6), *Nppb* (n = 8), and *Myh7* (n = 6)) was determined by RT-PCR. **P < 0.01 and ***P < 0.001, Student’s *t*-test. (**C**,**D**) Representative western blot images and the quantification of Sp1 and BNP in *pCMV-Sp1* and vector-transfected cells (n = 6). **P < 0.01 and ***P < 0.001, Student’s *t*-test. (**E**,**F**) Quantification of *Sp1* and *Nppb* mRNA levels in cells transfected with vector or *pCMV-Sp1* and treated with protocatechuic acid (10 μM, n = 6). ***P < 0.001; ^*###*^*P* < 0.001. (**G**) Sp1 and BNP protein expression in cells described in (**E**,**F)**, representative Western blot images. (**H**) Representative images of the cells transfected with vector or *pCMV-Sp1*, incubated with or without protocatechuic acid (10 μM), and then stained with anti-Sp1, Alexa Fluor 488 phalloidin, and DAPI. Sp1-positive cells (red); nuclei (blue); actin filaments (green). Scale bar = 50 μm. (**I**) Quantification of cell surface area (n = 30 cells). ***P < 0.001; ^*###*^*P* < 0.001*.* Data are presented as the mean ± S.E. Statistical analysis: one-way ANOVA followed by Bonferroni post hoc tests. Graphs were prepared using GraphPad 5.0.

To decipher the role of Sp1 in cardiac hypertrophy, we transfected H9c2 cells with *pCMV-Sp1*. As expected, the overexpression of Sp1 significantly enhanced mouse *Sp1* mRNA levels (Fig. 5A). Cardiac hypertrophy markers, such as *Nppa*, *Nppb*, and *Myh7*, were also upregulated by Sp1 overexpression (Fig. 5B). Western blotting confirmed that the protein levels of BNP were significantly increased in H9c2 cells (Fig. 5C,D). Furthermore, Sp1 overexpression induced *Sp1* and *Nppb* expression levels, while protocatechuic acid treatment reversed this effect (Fig. 5E,F). Western blot analysis showed similar results (Fig. 5G). Next, to evaluate the effect of Sp1 overexpression on cardiomyocyte phenotype, *pCMV-Sp1*-transfected cells were double-stained using anti-Sp1 antibody and Alexa Fluor phalloidin 488, and the cell size of Sp1-positive cells (red) was measured. As shown in Fig. 5H,I, Sp1 overexpression significantly increased the area of the cells; however, protocatechuic acid treatment reversed this effect.

### Protocatechuic acid reduced isoproterenol- or Sp1 overexpression-induced PKCγ expression

**Figure 6:**
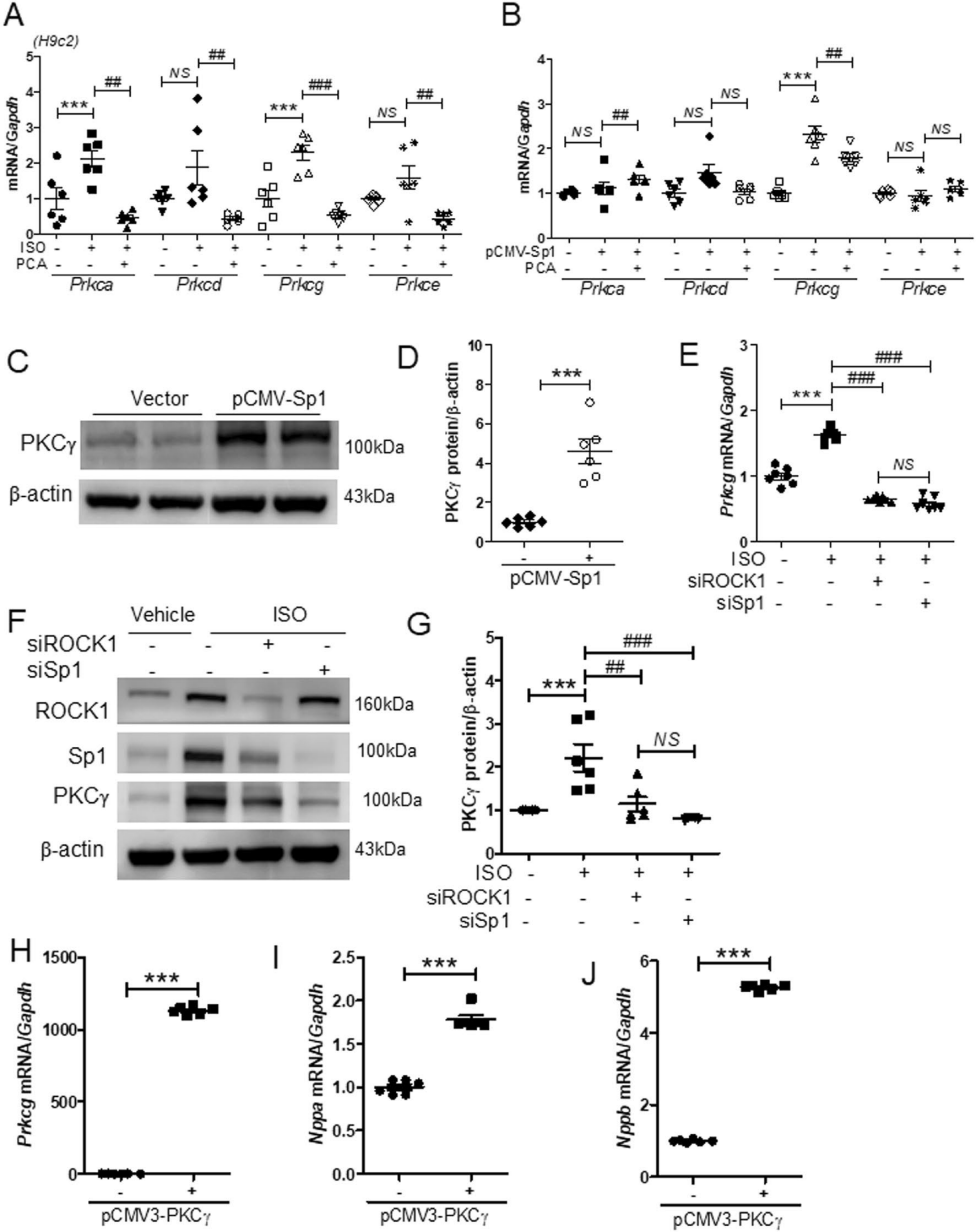
Protocatechuic acid attenuates isoproterenol- or Sp1 overexpression-induced PKCγ expression. (**A**) The quantification of PKC isoforms in H9c2 cells treated with isoproterenol (10 μM) in the presence or absence of protocatechuic acid (10 μM) for 6 h (n = 6). ***P < 0.001; ^*##*^*P* < 0.01 and ^*###*^*P* < 0.001; *NS* not significant. Data are presented as the mean ± S.E. Statistical analysis: one-way ANOVA followed by Bonferroni post hoc tests. (**B**) The quantification of PKC isoforms in H9c2 cells transfected with *pCMV-Sp1* and treated with protocatechuic acid (10 μM, n = 6). ***P < 0.001; ^*##*^*P* < 0.01; *NS* not significant. Data are presented as the mean ± S.E. Statistical analysis: one-way ANOVA followed by Bonferroni post hoc tests. (**C**) Representative western blots (the same blots as presented in Fig. 5C) of *pCMV-Sp1-*transfected cells; β-actin was used as a loading control. (**D**) Quantification of western blots presented in (**C**, n = 6). ***P < 0.001, Student’s *t*-test*.* (**E − G**) H9c2 cells were transfected with control, ROCK, or Sp1 siRNA for 24 h, serum starved, and then treated with isoproterenol (10 μM). (**E**) The mRNA levels of *Prkcg* were determined by RT-PCR (n = 7). ***P < 0.001; ^*###*^*P* < 0.001; *NS* not significant. Data are presented as the mean ± S.E. Statistical analysis: one-way ANOVA followed by Bonferroni post hoc tests. (**F**,**G**) Representative western blot images and quantification of PKCγ (n = 6). ***P < 0.001; ^*##*^*P* < 0.01 and ^*###*^*P* < 0.001; *NS* not significant*.* Data are presented as the mean ± S.E. Statistical analysis: one-way ANOVA followed by Bonferroni post hoc tests. (**H − J**) mRNA levels of *Prkcg*, *Nppa*, and *Nppb* in *pCMV3-N-GFPSpark-PKCγ*-transfected cells (n = 6). ***P < 0.001, Student’s *t*-test. Graphs were prepared using GraphPad 5.0.

It has been shown that the kinases that belong to the PKC family are involved in the regulation of cardiac hypertrophy^11^. Furthermore, PKC isoforms are reported to have distinct localization and function^35^. To evaluate the role of PKC family in our model system, we measured the expression of PKC isoforms in H9c2 cells. The mRNA levels of *Prkca* and *Prkcg* were increased in response to isoproterenol and decreased by protocatechuic acid treatment (Fig. 6A); *Prkcd* and *Prkce* levels were not significantly affected by isoproterenol. To further evaluate whether the expression of PKC isoforms could be affected by Sp1 overexpression, H9c2 cells were transfected with *pCMV* or *pCMV-Sp1* and then treated with vehicle or protocatechuic acid. Sp1 overexpression significantly increased the expression levels of *Prkcg* mRNA; however, the levels were reduced by protocatechuic acid treatment (Fig. 6B). Other PKC isoforms were not affected by Sp1 overexpression (Fig. 6B). Similar to gene expression results, the protein levels of PKCγ were also increased (Fig. 6C,D). To determine whether PKCγ is regulated by ROCK1 and Sp1, siRNA experiments were performed. The knockdown of ROCK1 or Sp1 significantly suppressed isoproterenol-induced PKCγ mRNA and protein levels (Fig. 6E–G). At the same time, Sp1 siRNA did not affect isoproterenol-induced ROCK1 protein levels (Supplementary Fig. 4A), while ROCK1 siRNA decreased isoproterenol-induced Sp1 protein levels (Supplementary Fig. 4B). In the absence of isoproterenol stimulation, Sp1 siRNA transfection did not alter *Rock1* mRNA levels (Supplementary Fig. 5A), ROCK1 siRNA reduced *Sp1* mRNA levels (Supplementary Fig. 5B), while *Prkcg* mRNA levels were significantly downregulated by both ROCK1 siRNA and Sp1 siRNA (Supplementary Fig. 5C). Next, we evaluated the role of PKCγ in cardiac hypertrophy. As expected, PKCγ overexpression increased *Prkcg* mRNA levels (Fig. 6H); however, it also upregulated the mRNA levels of *Nppa* and *Nppb* (Fig. 6I,J), but had no effect on *Rock1* and *Sp1* expression levels (Supplementary Fig. 6).

### Knockdown of PKCγ reduced isoproterenol-induced cardiac hypertrophy

**Figure 7:**
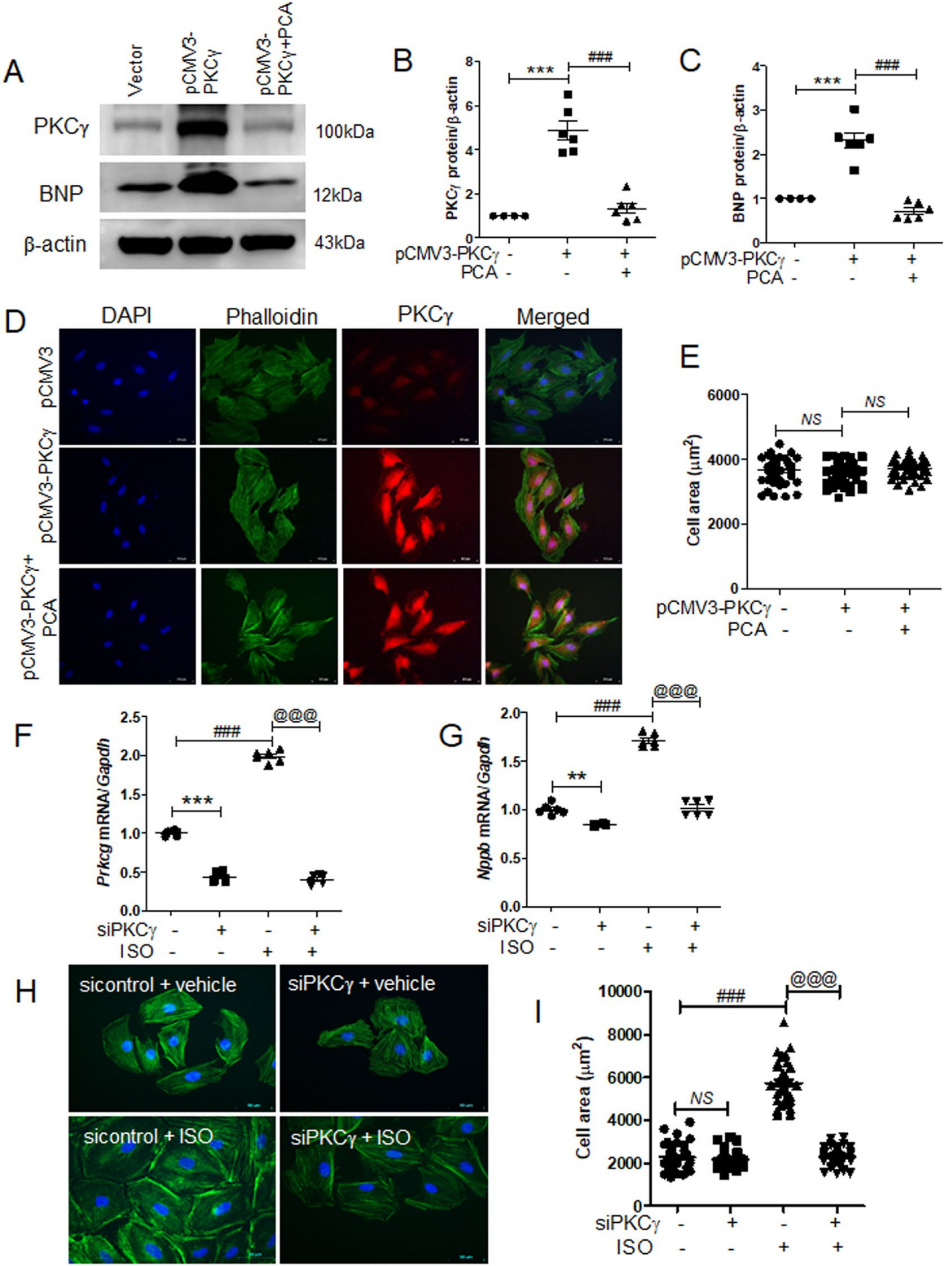
PKCγ knockdown reduces isoproterenol-induced cardiac hypertrophy in H9c2 cells. (**A‒E**) H9c2 cells were transfected with empty vector or *pCMV3-N-GFPSpark-PKCγ* and then treated with protocatechuic acid (10 μM). (**A**) Representative western blot images showing PKCγ and BNP protein levels in *pCMV3-N-GFPSpark-PKCγ*-transfected cells. β-actin was used as a loading control. (**B**,**C**) Quantification of PKCγ and BNP protein levels (n = 6). ***P < 0.001; ^*###*^*P* < 0.001. (**D**,**E**) Representative images of cells transfected with *pCMV3-N* vector or *pCMV3-N-GFPSpark-PKCγ*, incubated with or without protocatechuic acid (10 μM), and then stained with anti-PKCγ antibody, Alexa Fluor 488 phalloidin, and DAPI. PKCγ-positive cells (red); nuclei (blue); actin filaments (green). Scale bar = 50 μm. (**F**,**G**) H9c2 cells were transfected with control or PKCγ siRNA, serum starved overnight, and then treated with isoproterenol (10 μM). The mRNA levels of *Prkcg* and *Nppb* were determined by RT-PCR (n = 6). **P < 0.01 and ***P < 0.001; ^*###*^*P* < 0.001; ^*@@@*^*P* < 0.001. (**I**) Merged images of H9c2 cells transfected with PKCγ siRNA, incubated with or without isoproterenol (10 μM) for 24 h, and then stained with Alexa Fluor 488 phalloidin (green) and DAPI (blue). (**J**) Cell size was quantified by measuring the cell surface area of Alexa Fluor 488 phalloidin-stained cells (n = 40 cells). Scale bar = 50 μm. ^*###*^*P* < 0.001; ^*@@@*^*P* < 0.001; *NS* not significant. Data are presented as the mean ± S.E. Statistical analysis: one-way ANOVA followed by Bonferroni post hoc tests. Graphs were prepared using GraphPad 5.0.

To investigate the effect of protocatechuic acid on PKCγ-induced cardiac hypertrophy, H9c2 cells were transfected with *pCMV3-N-vector* or *pCMV3-N-GFPSpark-PKCγ* and then treated with protocatechuic acid. As expected, PKCγ overexpression increased endogenous PKCγ and BNP protein levels (Fig. 7A), while protocatechuic acid treatment reversed these effects (Fig. 7B,C). However, PKCγ overexpression did not affect the size of cardiomyocytes (Fig. 7D,E). To further investigate the connection between PKCγ and cardiac hypertrophy, PKCγ expression was downregulated using siRNA. PKCγ siRNA transfection reduced *Prkcg* mRNA levels (Fig. 7F), as well as isoproterenol-induced *Prkcg* and *Nppb* mRNA levels (Fig. 7F,G). Furthermore, the knockdown of PKCγ significantly decreased the isoproterenol-induced cell size (Fig. 7H,I).

### Downregulation of ROCK1 and Sp1 expression by protocatechuic acid was not regulated at the transcriptional level

**Figure 8:**
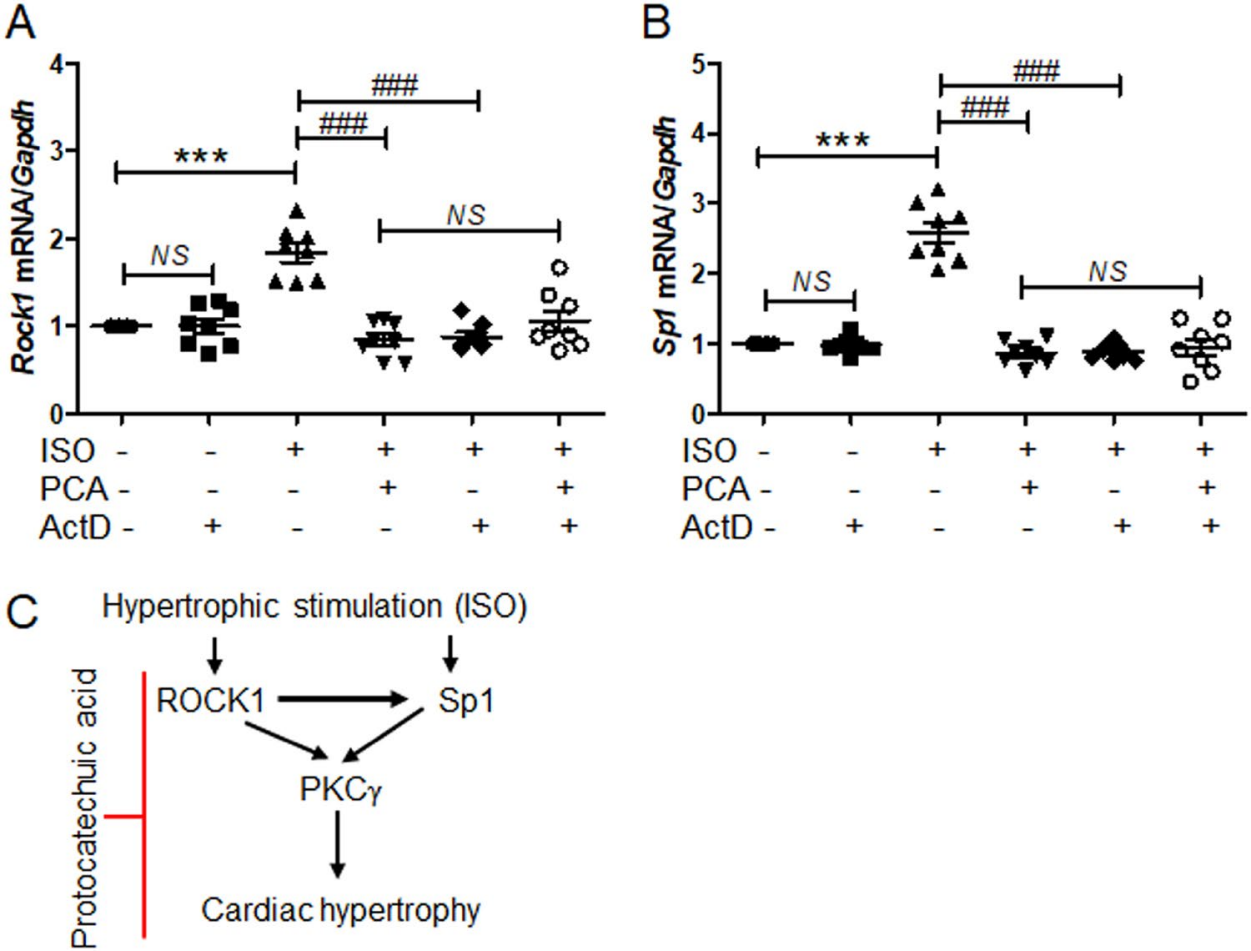
Protocatechuic acid does not regulate the expression of ROCK1 and Sp1 at the transcriptional level. (**A**,**B**) H9c2 cells were cultured with isoproterenol (10 μM) and then incubated with protocatechuic acid (10 μM) in the presence or absence of actinomycin D (2.5 μg/ml) for 6 h. The mRNA levels of *Rock1* and *Sp1* were quantified using RT-PCR and normalized to *Gapdh*. Data are presented as the mean ± S.E. Statistical analysis: one-way ANOVA followed by Bonferroni post hoc tests. ***P < 0.001; ^*###*^*P* < 0.001; NS, not significant. Graphs were prepared using GraphPad 5.0. (**C**) Schematic diagram of proposed mechanisms involved in isoproterenol-induced cardiac hypertrophy. Isoproterenol increases the expression of ROCK1, Sp1, and PKCγ. Sp1 increases the size of cardiomyocytes and induces PKCγ expression. Our results suggest that PKCγ is a downstream target of ROCK1 and Sp1. Protocatechuic acid treatment suppresses isoproterenol-induced cardiac hypertrophy via the downregulation of ROCK1–Sp1–PKCγ axis.

To investigate the mechanism of ROCK1 and Sp1 regulation by protocatechuic acid, H9c2 cells were treated with isoproterenol and then incubated with vehicle or protocatechuic acid in the presence or absence of actinomycin D, a well-characterized transcription inhibitor. In the absence of isoproterenol stimulation, actinomycin D treatment did not affect the expression of *Rock1* and *Sp1* mRNA. As expected, the expression of *Rock1* and *Sp1* induced by isoproterenol was decreased by protocatechuic acid. Furthermore, actinomycin D treatment also significantly reduced isoproterenol-induced expression of *Rock1* and *Sp1*; however, there was no additive inhibitory effect on *Rock1* and *Sp1* expression in cells treated with protocatechuic acid and actinomycin D (Fig. 8A,B).

## Discussion

Here, we showed, both in vitro and in vivo, that protocatechuic acid could be used as a potential treatment for cardiac hypertrophy. We demonstrated that protocatechuic acid attenuated cardiac hypertrophy by inhibiting the ROCK1–Sp1–PKCγ axis (Fig. 8C). Protocatechuic acid is a phenolic compound, structurally similar to gentisic acid^31^. We recently reported that gentisic acid inhibited cardiac hypertrophy in the mouse model of transverse aortic constriction (TAC)^31^. Gallic acid (3,4,5-trihydroxybenzoic acid), another phenolic compound with one more hydroxyl group than protocatechuic acid, also reduced isoproterenol-induced cardiac hypertrophy in mice^36^. Here we characterized protocatechuic acid-mediated inhibition of gross heart hypertrophy by evaluating echocardiographic parameters and cross-sectional area. Protocatechuic acid treatment reduced the isoproterenol-induced increase of cardiomyocyte surface area. Moreover, the isoproterenol-induced increase of left ventricular wall thickness was attenuated by protocatechuic acid. The activation of fetal genes, such as *Nppb* and *Myh7*, is characteristic of cardiac hypertrophy^37^. Here, we demonstrated that protocatechuic acid treatment decreased fetal gene expression both in vivo and in vitro.

Numerous vegetables and fruits, including green chicory, red chicory, black olives, and black raspberry, are rich in protocatechuic acid^38^. Protocatechuic acid is also a main metabolite of complex polyphenols, such as anthocyanins, which are widely distributed in the human diet. Protocatechuic acid is transformed to protocatechuic aldehyde by an aromatic carboxylic acid reductase^39^, another compound that was reported to protect against isoproterenol-induced cardiac hypertrophy in rats^40^.

Here we showed that the expression of ROCK1, Sp1, PKCα, and PKCγ was increased in response to isoproterenol stimulation in vitro. We hypothesized that the upregulation of these genes was linked to the development of cardiac hypertrophy. RhoA–ROCK pathway is involved in a wide range of biological functions, including contraction, migration, proliferation, differentiation, and apoptosis^41,42^. Activated Rho induces myofibrillar organization and ANP expression in myocytes^43^. The observations describing the role of ROCK1 in cardiac hypertrophy are contradictory. For example, TAC-induced cardiac hypertrophy and fibrosis were promoted in cardiac-specific ROCK1-deficient mice^44^. Global ROCK1 deletion mice, as well as hemizygous ROCK1^+/−^ mice, exhibited reduced cardiac fibrosis, and the development of cardiac hypertrophy was not affected after TAC or in response to angiotensin II treatment^45,46^. Shi et al.^47^ reported that in the transgenic model of Gαq overexpression-induced hypertrophy, ROCK1 deficiency improved the contractile function without reducing cardiac growth. However, our results clearly demonstrated that the knockdown of ROCK1 reduced the size of cardiomyocytes increased by isoproterenol treatment, suggesting that ROCK1 was involved in the pathogenesis of cardiac hypertrophy.

It has been reported that Sp1 levels are increased in right ventricular hypertrophy and in isoproterenol- or angiotensin II-stimulated cardiomyocytes^23,48,49^. In cardiomyocytes, Sp1 interacts with cardiac-specific transcription factor GATA4 and activates the ANP promoter^34^. Sp1 overexpression and knockdown experiments showed that Sp1 acted as an important mediator in the regulation of cardiac hypertrophy. Notably, Sp1 knockdown completely inhibited the isoproterenol-induced increase of cell surface area, as well as the expression of cardiac hypertrophic markers, in H9c2 cells, while Sp1 overexpression increased the expression of these genes. This is the first report demonstrating that Sp1 directly contributes to cardiac hypertrophy as determined by the measurement of cell size. Moreover, protocatechuic acid treatment reduced the Sp1 overexpression-induced increase of cell size and hypertrophic marker genes. These findings suggest that Sp1 plays a critical role in the regulation of cardiac growth.

The PKC family includes many different isoforms and is implicated in pathogenesis of cardiac hypertrophy^10^. Several studies reported conflicting results regarding the involvement of PKCβ in cardiac hypertrophy^15,16^. Furthermore, it was shown that the PKCα isoform induced cardiac hypertrophy in part through extracellular signal-regulated kinase 1/2, while PKCδ, PKCε, and PKCζ did not stimulate hypertrophic growth^12^. In the present study, we discovered that PKCγ played a role in cardiac hypertrophy. PKCγ induced the expression of hypertrophic genes, such as ANP and BNP, which were also increased by Sp1 overexpression. Moreover, PKCγ expression levels were significantly reduced by the knockdowns of ROCK1 and Sp1, either in the presence or absence of cardiac hypertrophic stimulation. These findings indicated that PKCγ was a new downstream target of Sp1 and ROCK1. Although PKCγ does not induce cardiac hypertrophy directly, our PKCγ siRNA experiments showed that it was indirectly involved in isoproterenol-induced cardiac hypertrophy. Furthermore, the isoproterenol-induced expression of ROCK1 and Sp1 was upregulated at the transcriptional level, whereas the downregulation of ROCK1 and Sp1 by protocatechuic acid was not mediated at the transcription level as determined by transcription inhibitor actinomycin D. However, the exact mechanisms involved in ROCK1 and Sp1 downregulation by protocatechuic acid still need to be elucidated.

So far, we have demonstrated that gallic acid, gentisic acid, and protocatechuic acid inhibited cardiac hypertrophy regardless of the number or location of hydroxyl group on benzoic acid. However, there was no direct comparative study of all three phenolic compounds.

In conclusion, we identified protocatechuic acid as an anti-hypertrophic phytochemical. Furthermore, ROCK1 and Sp1 are involved in the regulation the pathological hypertrophy state against beta-adrenergic agonist stimulus. Here we also show that Sp1 is a new pro-hypertrophic regulator. Our data indicate that ROCK1, Sp1, and PKCγ are involved in the development of cardiac hypertrophy and, therefore, could be used as new therapeutic targets for cardiac hypertrophy.

## Methods

### Reagents

Protocatechuic acid (3,4-dihydroxybenzoic acid; cat no. 37580), isoproterenol (cat no. I5627), and actinomycin D (cat no. A9415) were purchased from Sigma-Aldrich Co. (St. Louis, MO, USA). Anti-GAPDH (cat no. sc-32233), anti-β-actin (cat no. sc-47778), anti-BNP (cat no. sc-271185), anti-ROCK1 (cat no. sc-17794), anti-PKCγ (cat no. sc-166385), and anti-Sp1 (cat no. sc-17824) antibodies were from Santa Cruz Biotechnology (Dallas, TX, USA). Alexa Fluor 488 phalloidin (cat no. A12379) and Alexa Fluor 568 goat anti-mouse IgG (cat no. A11004) were purchased from Invitrogen (Eugene, OR, USA). Wheat germ agglutinin Alexa Fluor 488 conjugate (cat no. W11261) was purchased from ThermoFisher Scientific (Waltham, MA, USA). Human PKCγ cDNA clone expression plasmid was purchased from Sino Biological Inc. (cat no. HG112420ANG, Wayne, PA, USA).

### Ethical approval

This animal experiment was carried out in accordance with relevant guidelines and regulations, including compliance with the ARRIVE guidelines. All animal procedures were approved by the Animal Experimental Committee of Chonnam National University Medical School (CNUIACUC-20002) and were carried out according to the Guide for the Care and Use of Laboratory Animals (US National Institutes of Health Publications, 8th edition, 2011).

### Animal model of cardiac hypertrophy

Male CD-1 mice (age, 7 weeks; average weight ~ 33 g) were anesthetized by an intraperitoneal injection of ketamine (120 mg/kg) and xylazine (6.2 mg/kg), and cardiac hypertrophy was induced by isoproterenol infusion (25 mg/kg/day) using the osmotic minipump (Alzet). Mice were randomly divided into three following groups (n = 8/group): vehicle-treated sham group, isoproterenol-infused group, and isoproterenol-infused group with protocatechuic acid (100 mg/kg/day). Isoproterenol was dissolved in 0.1% ascorbic acid and 0.9% saline, while protocatechuic acid was dissolved in dimethyl sulfoxide (DMSO) and diluted using 0.9% saline. Both drugs were administered for 5 days: isoproterenol was continuously infused using an osmotic minipump (24 h/day), while protocatechuic acid was administered daily via intraperitoneal injection (total volume 400 μl). The mice were euthanized after 5 days.

### Echocardiography

Echocardiography was performed using a Vivid S5 echocardiography system (GE Healthcare, Chicago, IL, USA) with a 13-MHz linear array transducer. Mice were anesthetized by an intraperitoneal injection of tribromoethanol (Avertin; 114 mg/kg) before the procedure. M-mode (2-D guided) images and recordings were acquired from the long-axis view of the left ventricle at the level of the papillary muscles. The thickness of left ventricular posterior and interventricular septa was measured from the images, whereas the left ventricular end-diastolic diameter (LVEDd) and left ventricular end-systolic diameter (LVESd) were measured from the M-mode recordings.

### Histology

Mice were euthanized using a 100% grade CO_2_ for approximately 2–3 min. The hearts of mice were fixed with 4% paraformaldehyde and embedded in paraffin. The paraffin-embedded tissues were then cut into 3-µm sections, deparaffinized with xylene, and rehydrated in the series of graded ethanols. To measure the cross-sectional cardiomyocyte area, tissue sections were stained with hematoxylin and eosin (H&E) as previously described^50^. To evaluate cell morphology, sections were also stained with wheat germ agglutinin conjugated to Alexa Fluor 488 (1:200) as previously described^36^.

Digital images were obtained using a microscope (Nikon Eclipse 80*i* microscope, Tokyo, Japan) at a 400 × magnification. The cross-sectional area was quantified using the NIS Elements Software Version AR 3.0 (https://www.nikonmetrology.com/images/brochures/nis-elements-en.pdf,Nikon, Tokyo, Japan).

### Reverse transcription polymerase chain reaction (RT-PCR)

Total RNA was isolated from heart tissues using TRIzol reagent (Invitrogen/Life Technologies, Carlsbad, CA, USA) and 1 μg was reverse transcribed with TOPscript RT DryMIX (Enzynomics, Daejeon, South Korea). qRT-PCR was performed with a SYBR Green PCR kit (Enzynomics) and gene expression was quantified using the 2^-∆∆Ct^ method. The PCR primers used in this study are listed in Table 1.

**Table 1:** Primers for RT-PCR.

Gene	Primer sequence (5′ to 3′)
*Gapdh *(rat)	F: AACCCATCACCATCTTCCAGGAGC R: ATGGACTGTGGTCATGAGCCCTTC
*Nppa *(rat)	F: GCTCGAGCAGATCGCAAAAG R: GAGTGGGAGAGGTAAGGCCT
*Nppb *(rat)	F: GACGGGCTGAGGTTGTTTTA R: ACTGTGGCAAGTTTGTGCTG
*Myh7* (rat)	F: CCTCGCAATATCAAGGGAAA R: TACAGGTGCATCAGCTCCAG
*Rock1* (rat)	F: CTGGGAAGAAAGGGACATCA R: TTCAGGCACATCGTAGTTGC
*Sp1* (rat = mouse)	F: TCTGCAGCTACCCTGACTCC R: TAATTCCCATGTTGCTGGTG
*Prkcg* (rat)	F: TTGATGGGGAAGATGAGGAG R: CGGACGTGGTCTAAAAGGAG
*Prkca* (rat)	F: TTCCTGACCCCAAGAATGAG R: CATGAAGTCATTCCGTGTCG
*Prkcd* (rat)	F: AGCCTCTCCCTCTCTTCCAC R: CTTCATCTTCACGGCACAGA
*Prkce* (rat)	F: TGCTCCTGCTCTTCAATCCT R: TGTTGGTCTTCTGCTTGGTG
*Gapdh* (mouse)	F: GCATGGCCTTCCGTGTTCCT R: CCCTGTTGCTGTAGCCGTATTCAT
*Nppb* (mouse)	F: CTGAAGGTGCTGTCCCAGAT R: GTTCTTTTGTGAGGCCTTGG
*Myh7* (mouse)	F: GCATTCTCCTGCTGTTTCCT R: CCCAAATGCAGCCATCTC

### Western blotting

Total protein was extracted from heart tissues or cells using RIPA lysis buffer (150 mM NaCl, 1% Triton X-100, 1% sodium deoxycholate, 50 mM Tris–HCl at pH 7.5, 2 mM EDTA, 1 mM PMSF, 1 mM DTT, 1 mM Na_3_VO_4_, and 5 mM NaF) containing a protease inhibitor cocktail (Calbiochem/EMD Millipore, Billerica, MA, USA). Proteins were subjected to SDS-PAGE, transferred to polyvinylidene difluoride membranes, and then blocked with 5% skim milk in TBST buffer (20 mM Tris, 200 mM NaCl, and 0.04% Tween 20) for 1 h at 25 °C. The membranes were incubated with primary antibodies against BNP, Sp1, ROCK1, PKCγ, β-actin, or GAPDH overnight at 4 °C, followed by anti-rabbit or anti-mouse horseradish peroxidase-conjugated secondary antibodies (diluted 1:5,000) for 1 h at 25 °C. Protein bands were visualized using Immobilon western blotting detection reagents (EMD Millipore, Billerica, MA, USA). Bio-1D Software Version 15.01 (https://www.witec.ch/products/imaging-uv-systems/bio-1d-analysis-software, Vilber Lourmat, Eberhardzell, Germany) was used to quantify protein expression.

### Cell culture and cell size measurement

H9c2 cardiomyoblast cell line (catalog no. 21446; Korean Cell Line Bank, Seoul, Korea) was maintained in the Dulbecco’s Modified Eagle’s Medium containing 10% fetal bovine serum (FBS) in 5% CO_2_ incubator at 37 °C. Cells were seeded on coverslips at a density of 1 × 10^4^ cells/well, serum-starved overnight, and then treated with vehicle or protocatechuic acid (10 μM) in the presence or absence of isoproterenol (10 μM) for 24 h. Cells were fixed with paraformaldehyde, permeabilized with 0.2% Triton X-100, incubated with Alexa Fluor 488 phalloidin (1:200) for 45 min, and then stained with 4’,6-diamidino-2-phenylindole (DAPI). The cell size was measured using the NIS Elements Software Version AR 3.0 (https://www.nikonmetrology.com/images/brochures/nis-elements-en.pdf, Nikon, Tokyo, Japan).

### Sp1 or PKCγ transfection

To evaluate the expression of cardiac hypertrophic markers and signaling-related genes, H9c2 cells were transfected with empty vector, *pCMV-Sp1*, or *pCMV3-N-GFPSpark-PKCγ* using Plus and Lipofectamine reagents (Invitrogen, Waltham, MA, USA) for 24 h. For cell size measurements, H9c2 cells were transfected with empty vector, *pCMV-Sp1,* or *pCMV3-N-GFPSpark-PKCγ*, and then treated with vehicle or protocatechuic acid (10 μM) for 9 h. Cells were fixed with 2.7% paraformaldehyde for 30 min, permeabilized with 0.1% Triton X-100, blocked with goat serum for 1 h, and then incubated with the anti-Sp1 (1:200) or anti-PKCγ (1:200) antibody overnight at 4 °C. Next, cells were washed and incubated with Alexa Fluor 488 Phalloidin (1:200) for 1 h. The nuclei were counterstained with DAPI. Sp1-positive (red) or PKCγ-positive (red) cells were identified and the cell surface area of positive cells was measured using the NIS Elements Software Version AR 3.0 (https://www.nikonmetrology.com/images/brochures/nis-elements-en.pdf, Nikon, Tokyo, Japan).

### Cell viability

H9c2 cells were seeded in 24-well culture plates and then treated with 1, 10, and 100 μM concentrations of protocatechuic acid for 24 h. Next, the cells were incubated with a 3-(4,5-dimethylthiazol-2-yl)-2,5-diphenyltetrazolium bromide (MTT) solution for 2 h. The insoluble formazan crystals were dissolved using DMSO, and the absorbance was measured at 570 nm.

### Small interfering RNAs (siRNA) transfection

H9c2 cells were transfected with 100 nM control siRNA (cat no. SN-1003, Bioneer, South Korea), Sp1 siRNA (product name 1762865, Bioneer, South Korea), PKCγ siRNA (product name 24681, Bioneer, South Korea), or ROCK1 siRNA (cat no. L-095591-02-0005, Dharmacon Inc., Lafayette, CO, USA) using RNAiMAX reagent according to the manufacturer’s instructions. siRNA control sense: 5′-CCU ACG CCA CCA AUU UCG U-3′, siRNA control antisense: 5′-ACG AAA UUG GUG GCG UAG G-3′; siRNA Sp1 sense: 5′-GUG CAA AUC AAC AGA UCA U-3′, siRNA Sp1 antisense: 5′-AUG AUC UGU UGA UUU GCA C-3′; siRNA PKCγ sense: 5′-GUC CGA AUU UUA GGU CUC U-3′, siRNA PKCγ antisense: 5′-AGA GAC CUA AAA UUC GGA C-3′. The target sequences used for rat ROCK1 were pooled with no. 1: GCAAAGAGAGUGAUAUUGA, no. 2: CCAGGAAGGUUUAUGCUAU, no. 3: AUAGAAAGCUCCAACUAGA, no. 4: CCAAAGAUAUUGAACUACU.

### Statistical analysis

All data are expressed as the mean ± standard error (SE). The Student’s *t*-test was used to compare two groups. The differences between three or more groups were analyzed by one-way analysis of variance (ANOVA) followed by the Bonferroni post hoc test using the GraphPad Prism version 5 software (https://www.graphpad-prism.software.informer.com/5.0, GraphPad Software, La Jolla, CA, USA). Graphs were also prepared using GraphPad Prism version 5. *P* values < 0.05 were considered as statistically significant.

## Supplementary Information

Supplementary Figures.
