# Supplementary material for: Protocatechuic acid attenuates isoproterenol-induced cardiac hypertrophy via downregulation of ROCK1–Sp1–PKCγ axis
Source: Sci Rep. 2021 Aug 30;11:17343. doi: 10.1038/s41598-021-96761-2 (PMC8405624; doi:10.1038/s41598-021-96761-2)

SUPPORTING INFORMATION

Protocatechuic acid attenuates isoproterenol-induced cardiac hypertrophy *via* downregulation of ROCK1-Sp1-PKCγ axis

Liyan Bai[1,2], Hae Jin Kee[1,2*], Xiongyi Han[1,2], Tingwei Zhao[1,2], Seung-Jung Kee[3], and Myung Ho Jeong[1,2,4*]

[1]Heart Research Center of Chonnam National University Hospital, Gwangju 61469, Republic of Korea

[2]Hypertension Heart Failure Research Center, Chonnam National University Hospital, Gwangju 61469, Republic of Korea

[3]Department of Laboratory Medicine, Chonnam National University, Medical School and Hospital, Gwangju 61469, Republic of Korea

[4]Department of Cardiology, Chonnam National University Medical School, Gwangju 61469, Republic of Korea

*Correspondence to

Hae Jin Kee, PhD

Heart Research Center of Chonnam National University Hospital, 42 Jebong-ro, Dong-gu, Gwangju 61469, Republic of Korea; Tel: +82-62-220-5823; Fax: +82-62-228-4227; E-mail: sshjkee@empas.com

Myung Ho Jeong, MD, PhD, FACC, FAHA, FESC, FSCAI, FAPSIC

Director of Heart Research Center Nominated by Korea Ministry of Health and Welfare, Chonnam National University Hospital, 42 Jebong-ro, Dong-gu, Gwangju 61469, Republic of Korea; Tel: +82-62-220-6243; Fax: +82-62-228-7174; E-mail: myungho@chollian.net

Supplementary Figures

Figure S1. Protocatechuic acid regulates left ventricular remodeling.

(**A–C**) Echocardiographic parameters were determined in sham, isoproterenol, and isoproterenol + protocatechuic acid-treated mice. LVIDd, left ventricular internal diameter at diastole; LVIDs, left ventricular internal diameter at systole; EF, ejection fraction (%). $^{**}P<0.01$; $^{\#}P<0.05$; NS, not significant. Graphs were prepared using GraphPad 5.0.

Figure S2. Knockdown of ROCK1 reduces the protein expression levels of ANP and BNP.

H9c2 cells were transfected with control or ROCK1 siRNA, serum-starved, and then treated with isoproterenol (10 µM). The protein levels of ROCK1 (**A**), ANP (**B**), and BNP (**C**) were quantified using the ImageJ software. $^{**}P<0.01$ and $^{***}P<0.001$; $^{\#}P<0.05$, $^{\#\#}P<0.01$, and $^{\#\#\#}P<0.001$. Graphs were prepared using GraphPad 5.0.

Figure S3. Knockdown of ROCK1 does not affect the cell size in the absence of isoproterenol.

H9c2 cells were transfected with control or ROCK1 siRNA and serum-starved. (**A**) Cells were stained with Alexa Fluor 488 phalloidin and counterstained with DAPI. Merged images are shown. Scale bar = 50 µm.

(**B**) Quantification of cell area. Cell area was measured using the NIS Elements Software Version AR 3.0 (https://www.nikonmetrology.com/images/brochures/nis-elements-en.pdf). Graphs were prepared using GraphPad 5.0.

Figure S4. Knockdown of ROCK1 or Sp1 reduces isoproterenol-induced Sp1 or ROCK1 protein levels.

H9c2 cells were transfected with control, ROCK1, or Sp1 siRNA, serum-starved, and then treated with isoproterenol. The protein levels of ROCK1 (**A**) and Sp1 (**B**) were quantified using the ImageJ software. *$P<0.05$ and ***$P<0.001$; ##$P<0.01$ and ###$P<0.001$; NS, not significant. Graphs were prepared using GraphPad 5.0.

Figure S5. PKCγ is a downstream target of ROCK1 and Sp1.

H9c2 cells were transfected with control, ROCK1, or Sp1 siRNA for 2 days. (**A**−**C**) The mRNA levels of *Rock1*, *Sp1*, and *Prkcg* were determined by RT-PCR. ***$P<0.001$; ###$P<0.001$; NS, not significant. Graphs were prepared using GraphPad 5.0.

Figure S6. PKCγ overexpression does not affect *Rock1* and *Sp1*.

H9c2 cells were transfected with pCMV3-N vector or pCMV3-N-GFPSpark-PKCγ for 24 h. *Rock1* and *Sp1* mRNA levels were determined by RT-PCR. NS; not significant. Graphs were prepared using GraphPad 5.0.

Supplementary Figure 1

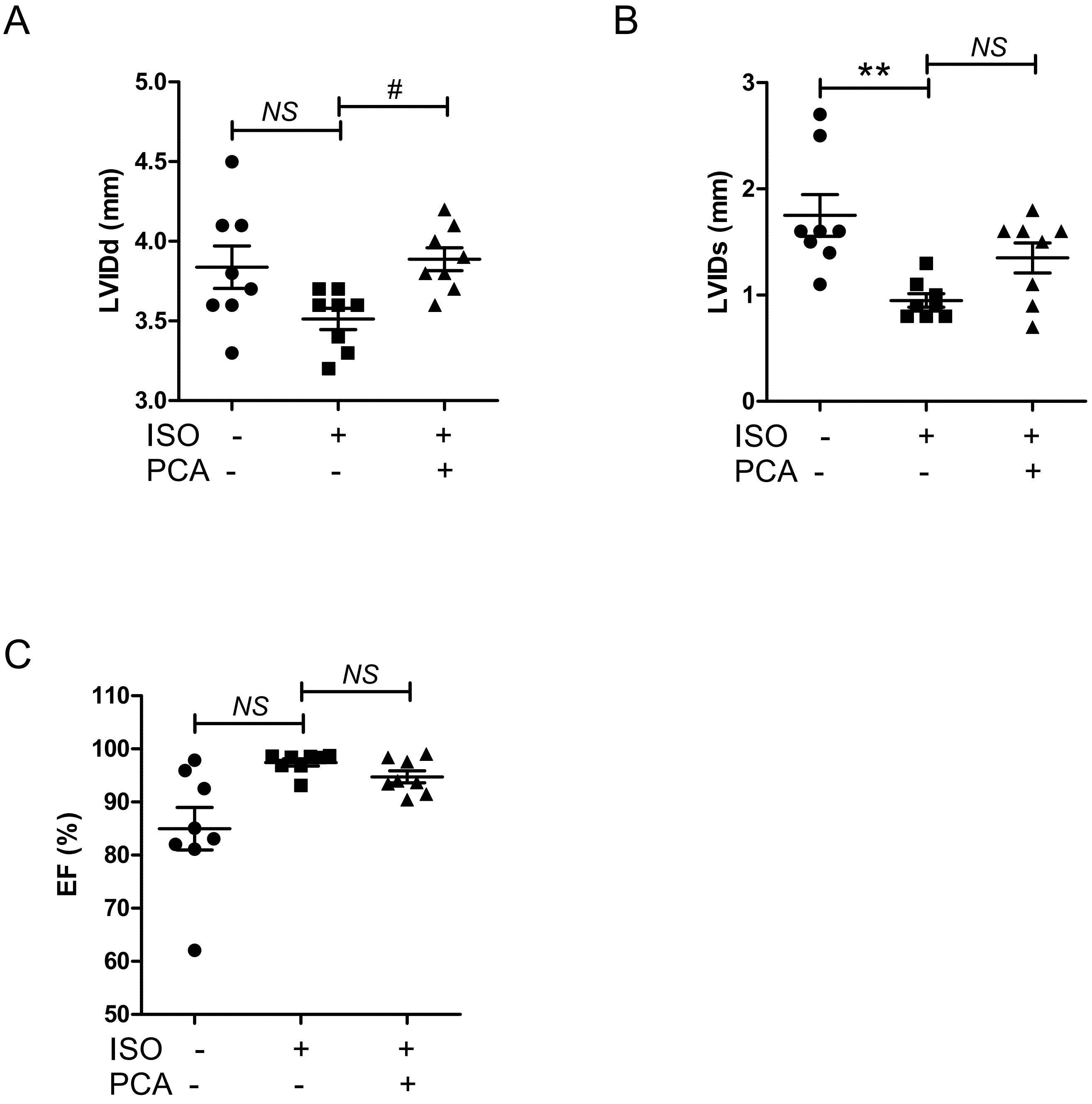

Supplementary Figure 2

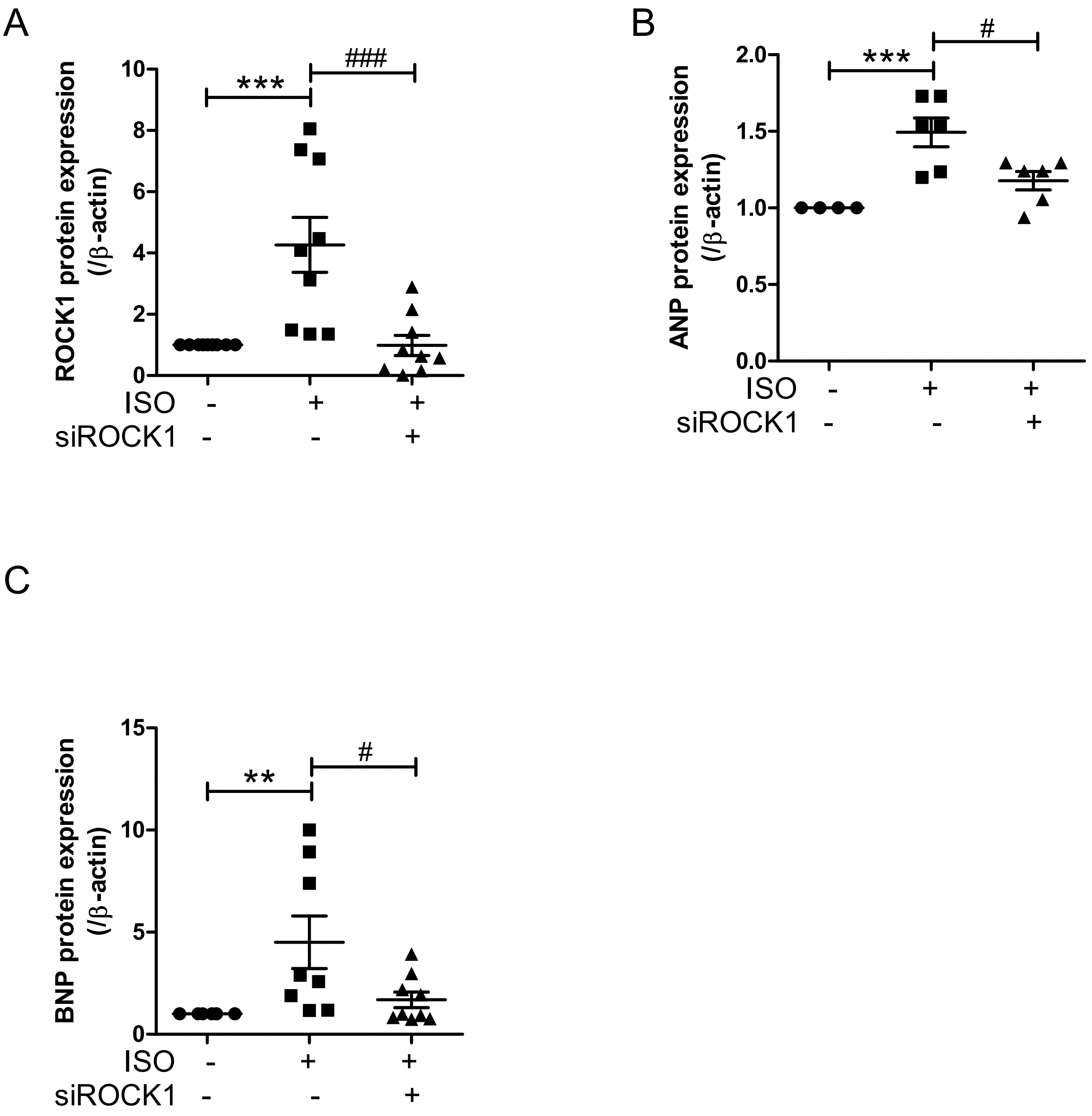

Supplementary Figure 3

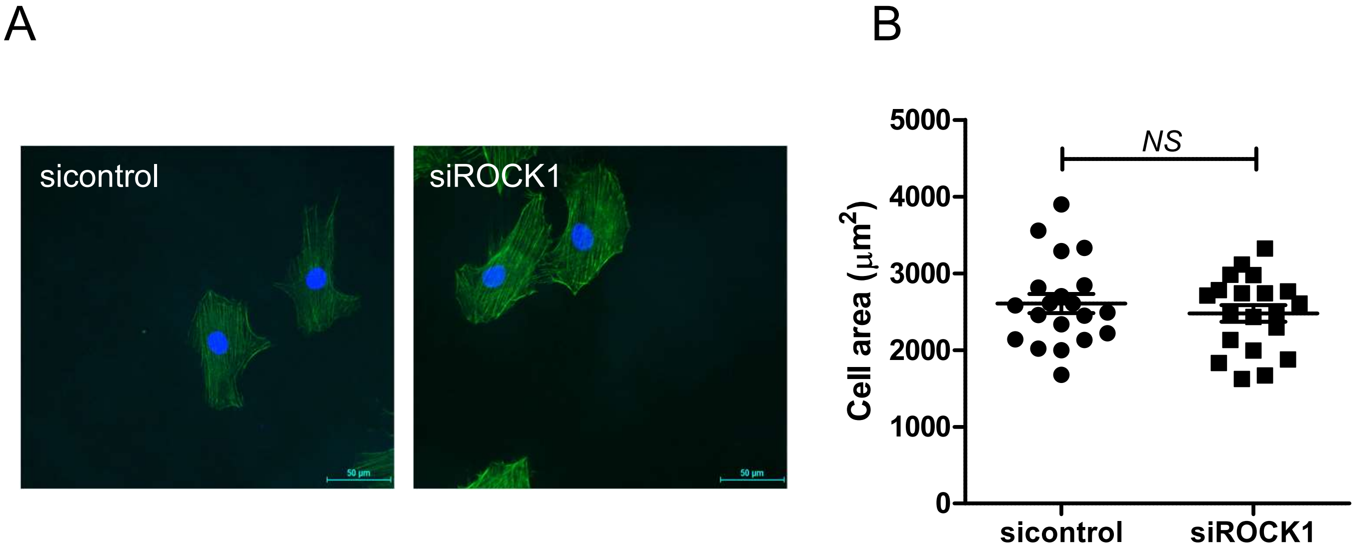

Supplementary Figure 4

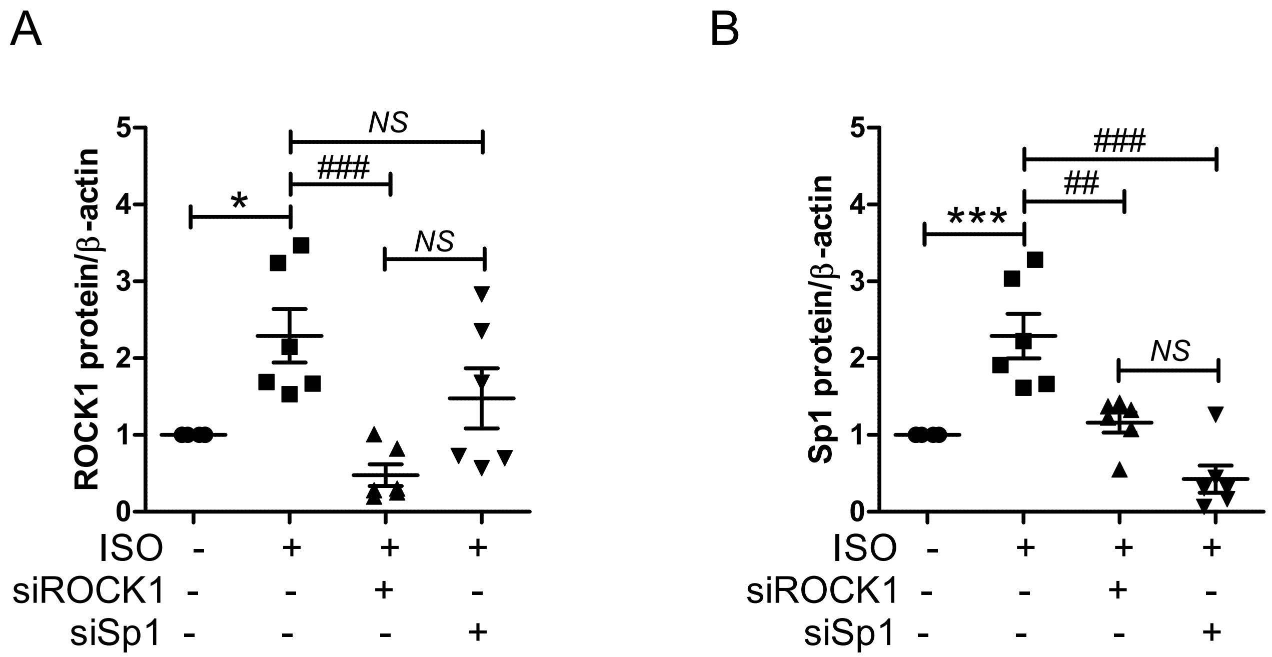

Supplementary Figure 5

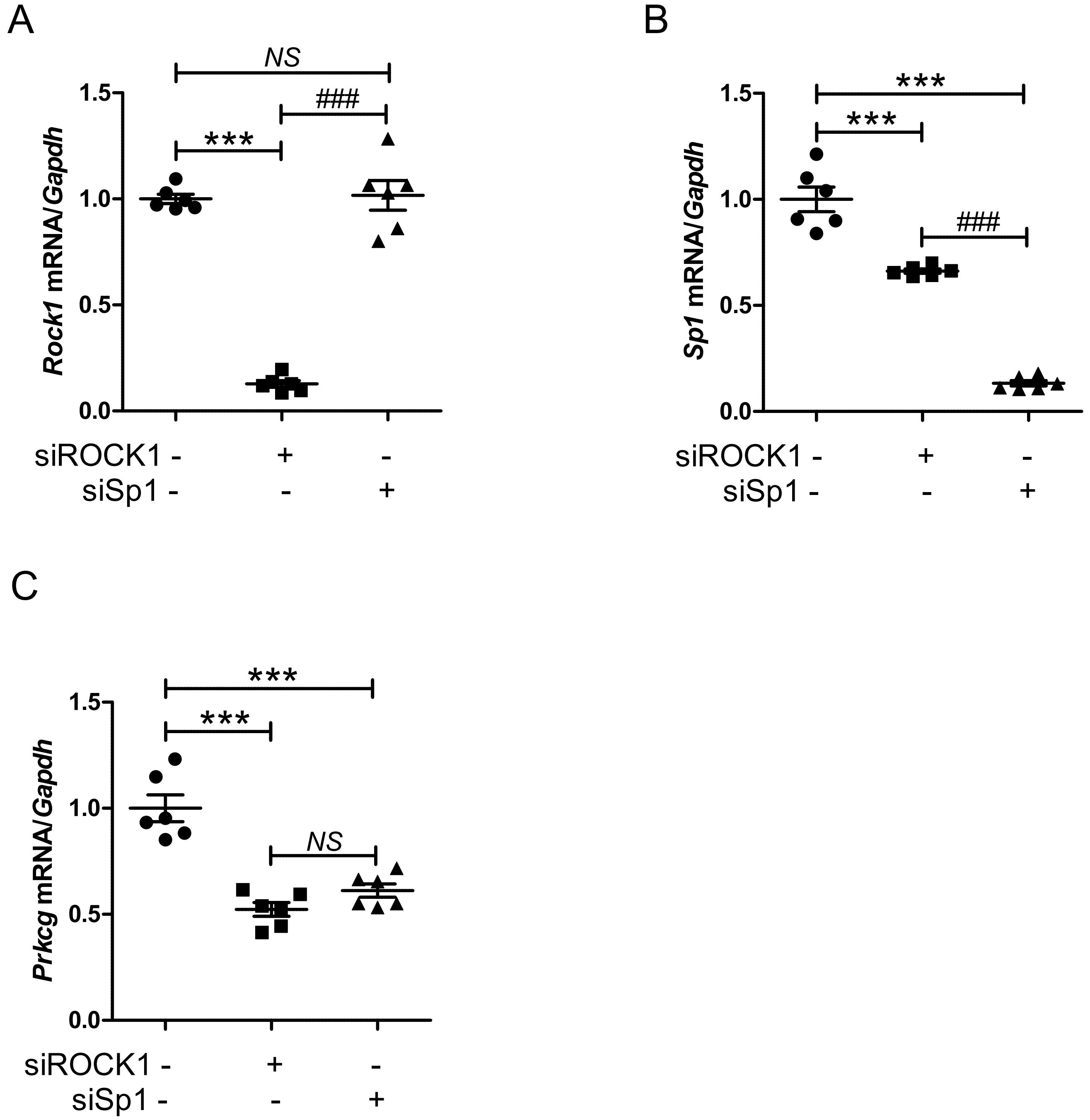

Supplementary Figure 6

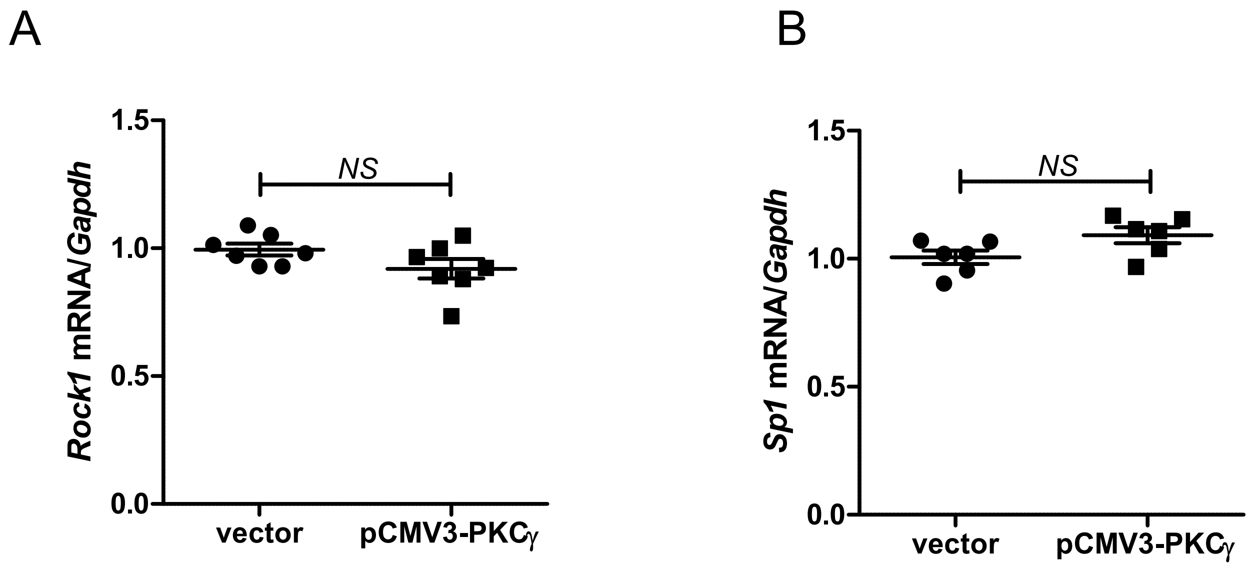

Full blots

Figure 1G WB original blots (right)

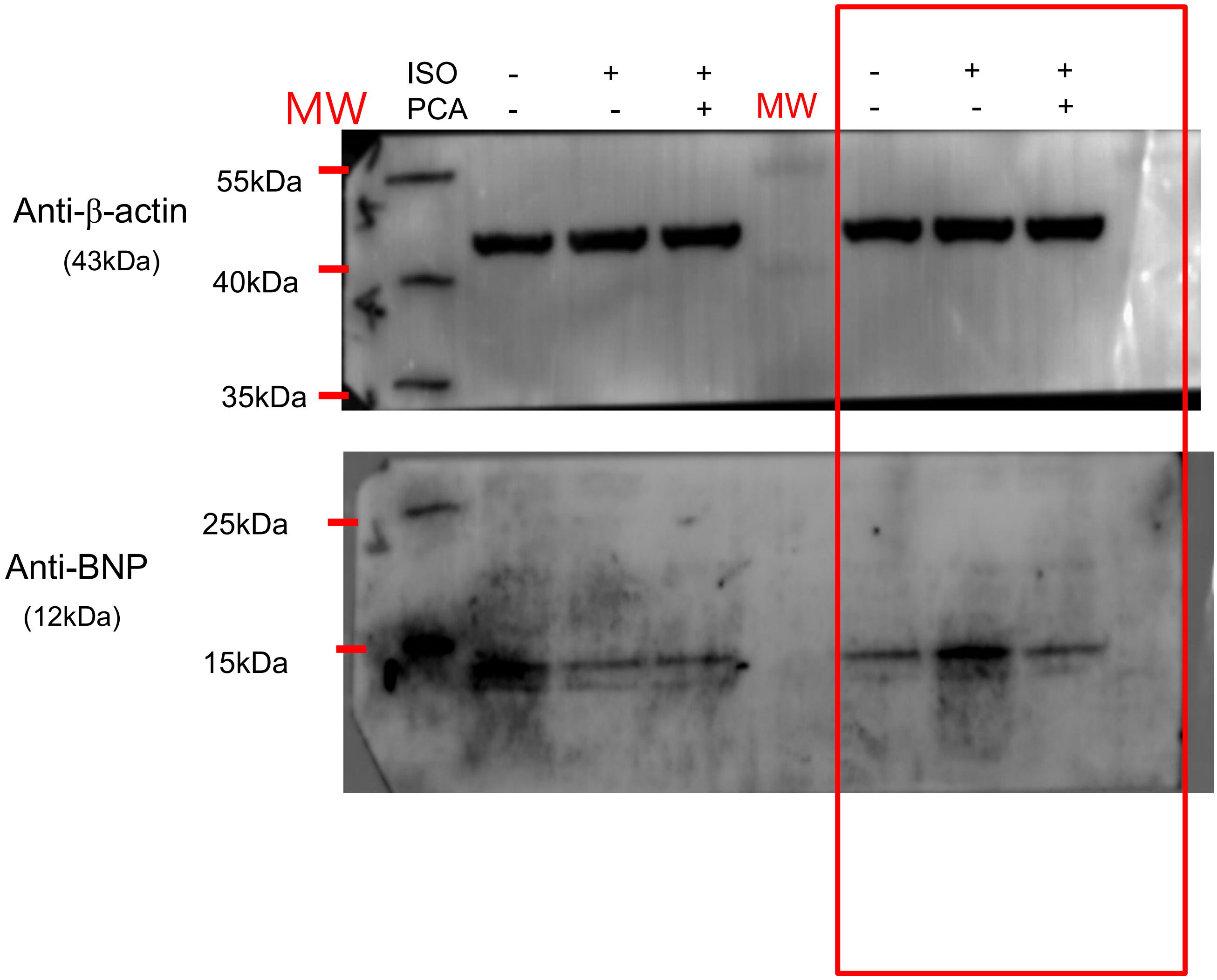

Figure 2K original blot

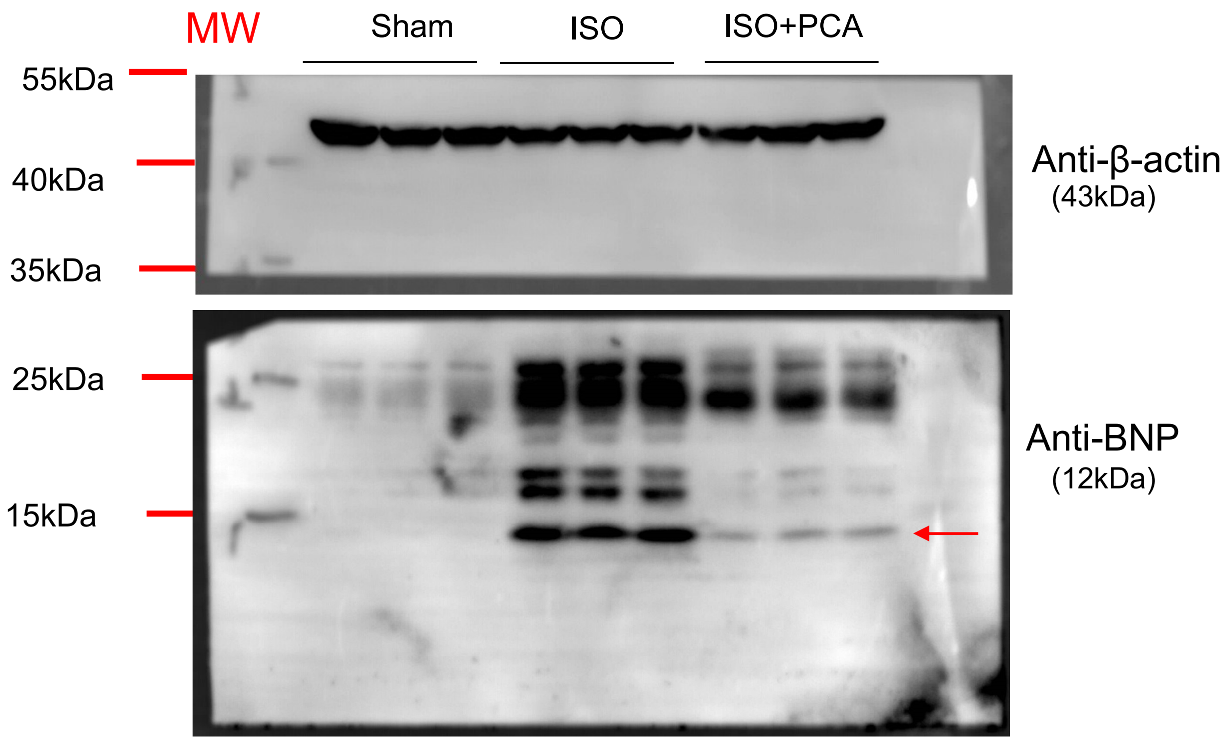

Figure 3A original blot

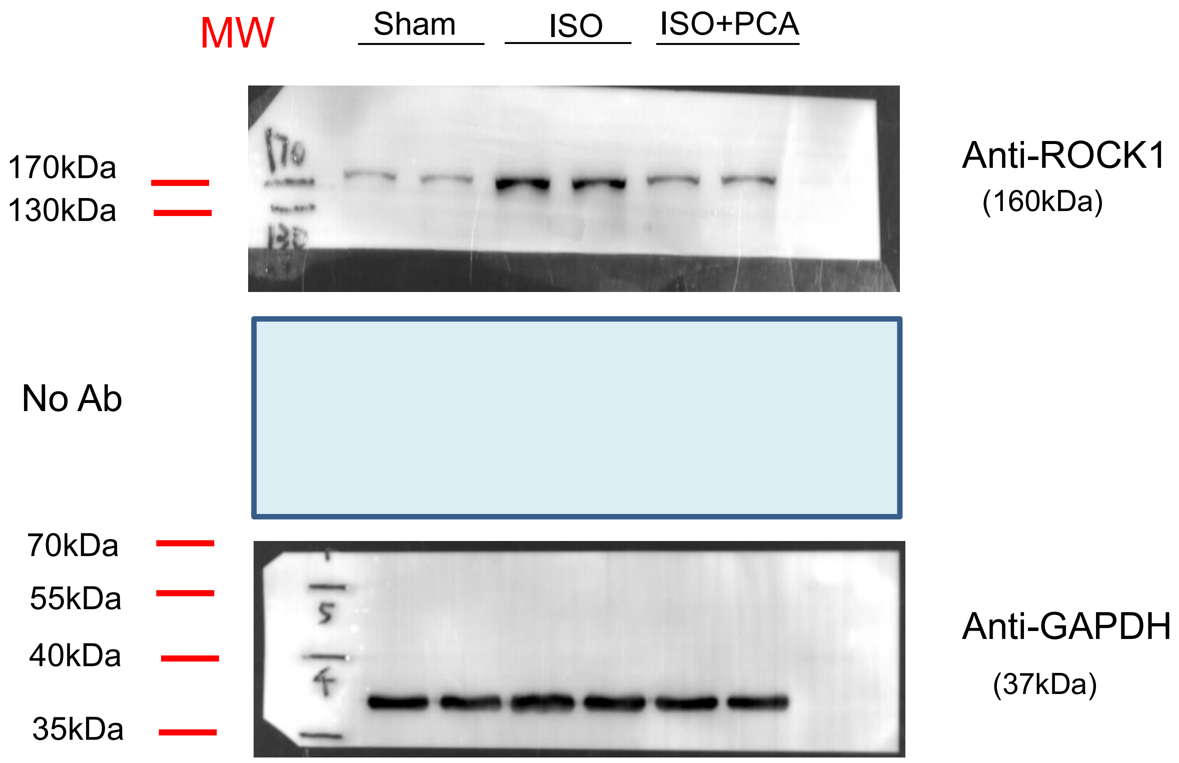

Figure 3D WB original blots (left)

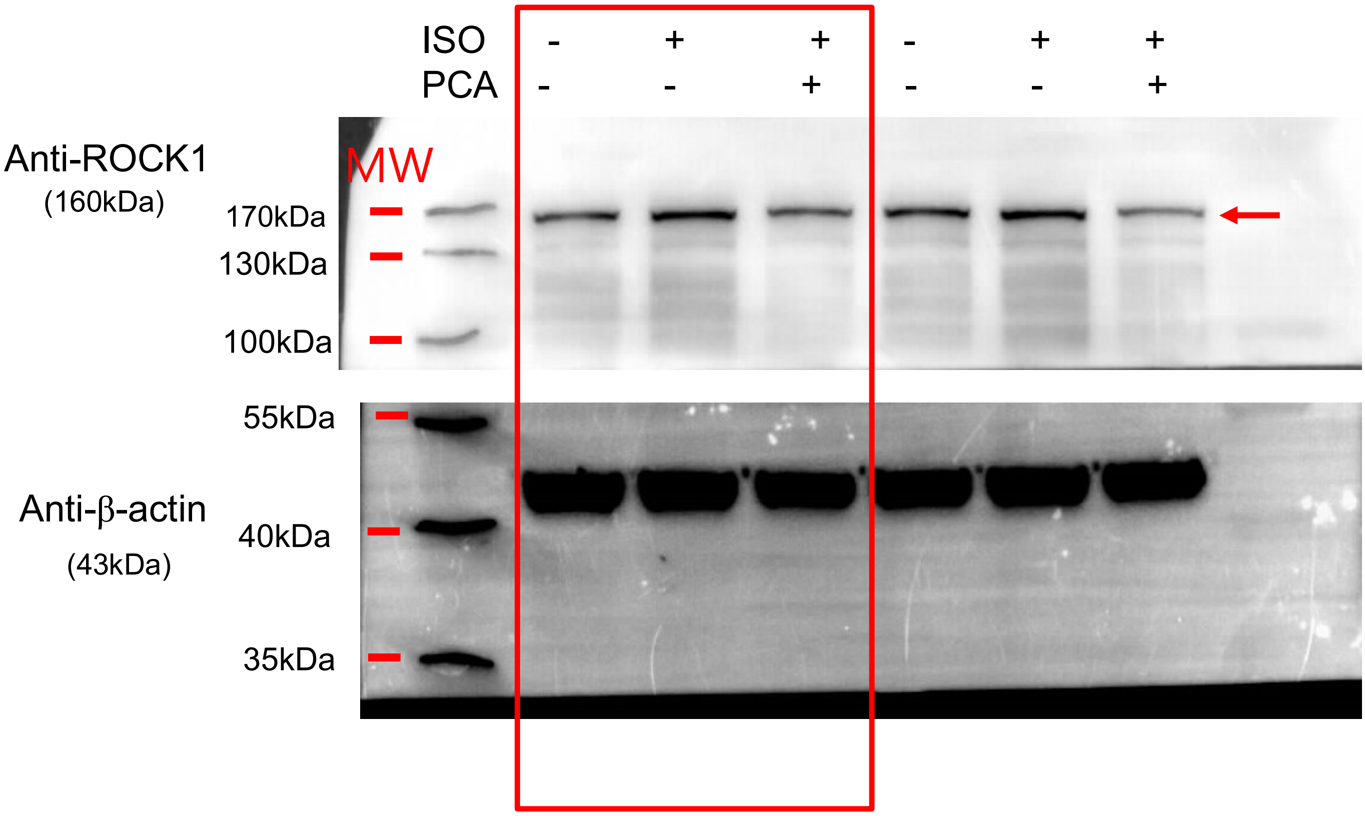

Figure 3H original blot

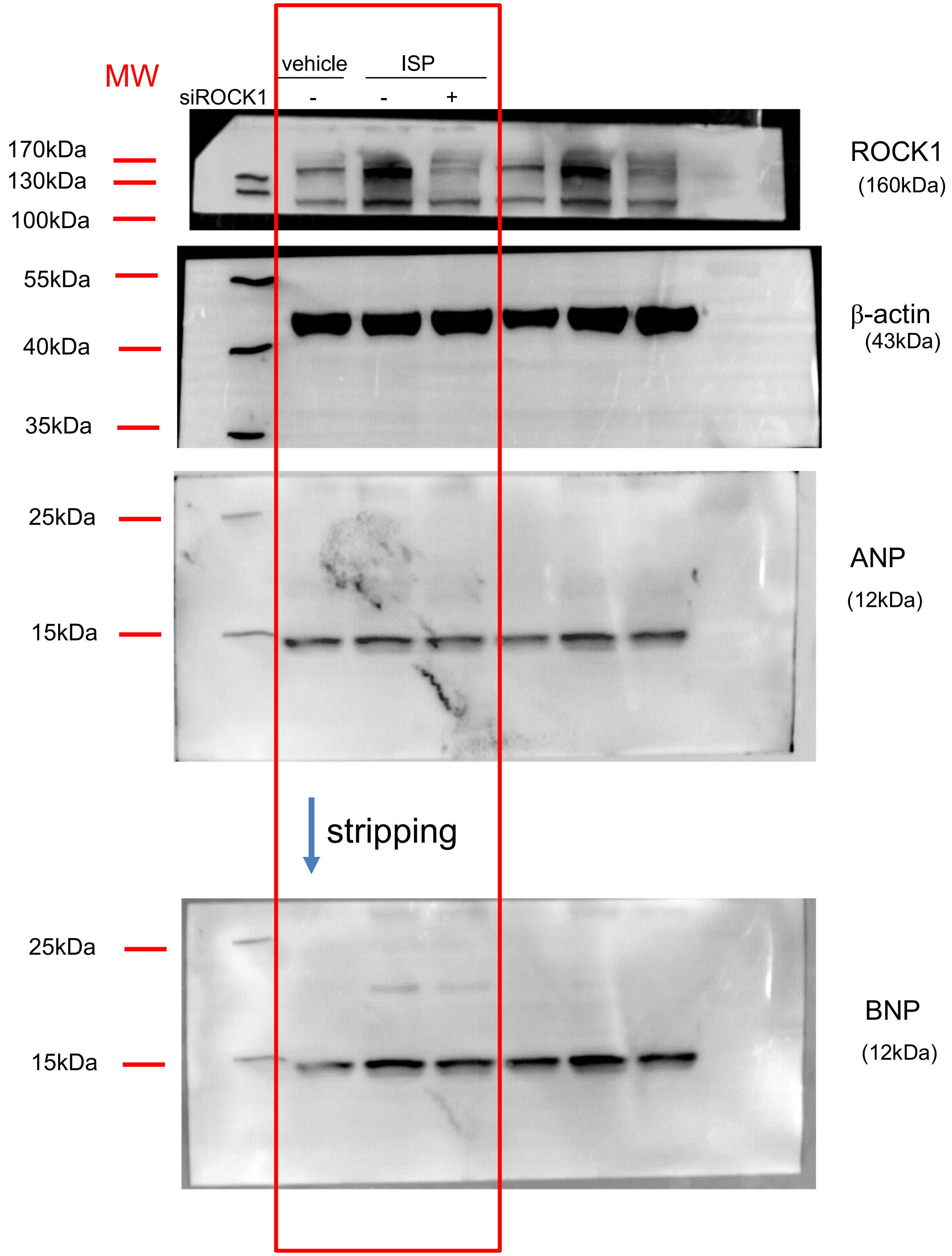

Figure 4C original blot

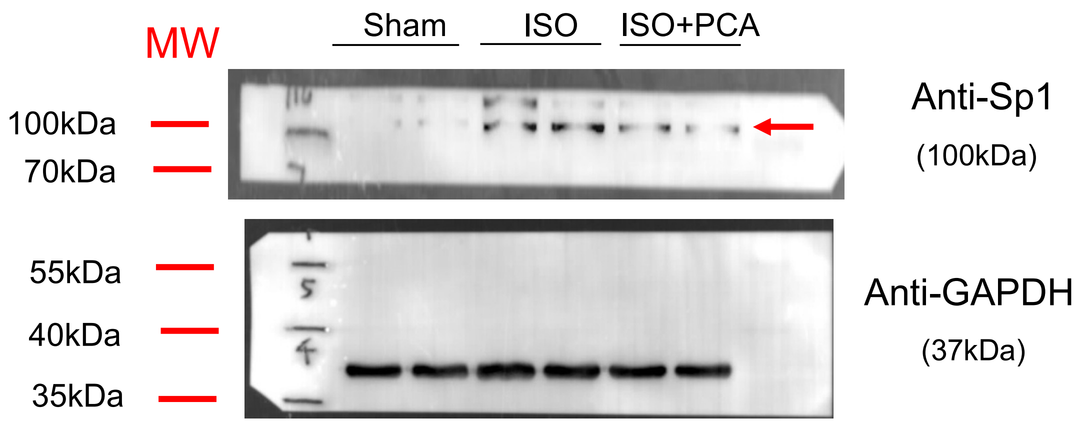

Figure 5C original blot

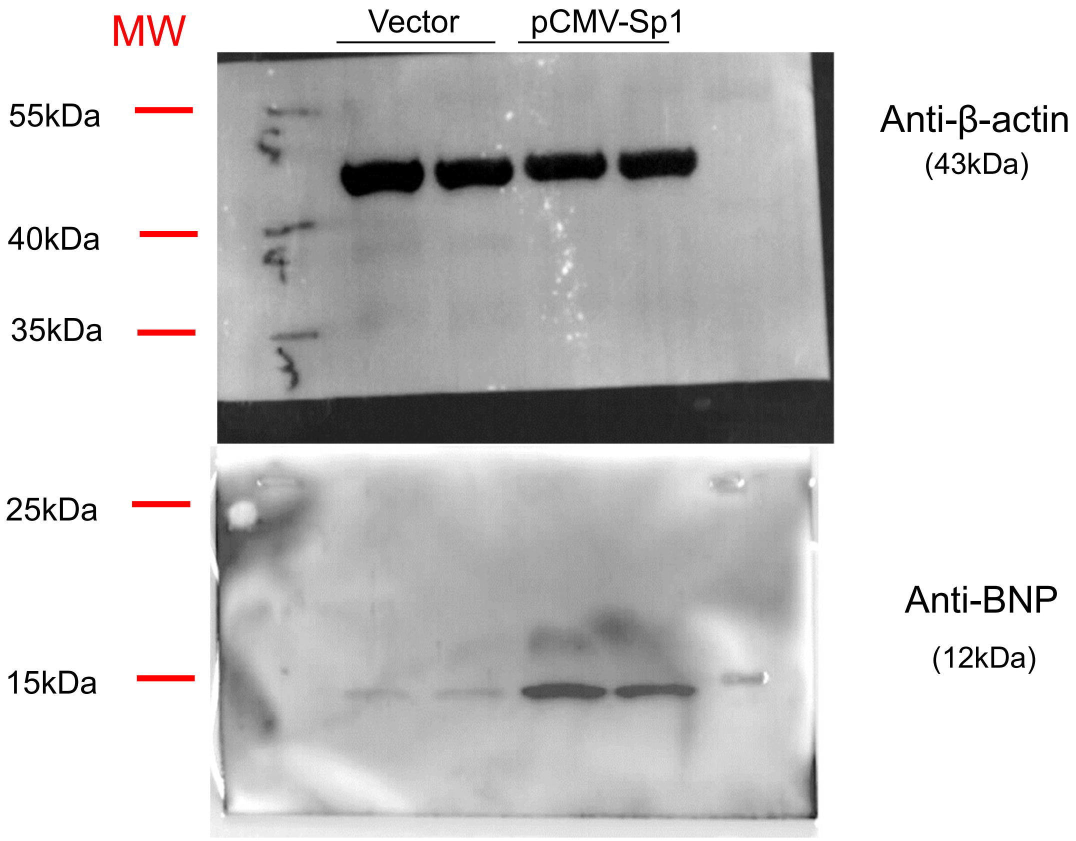

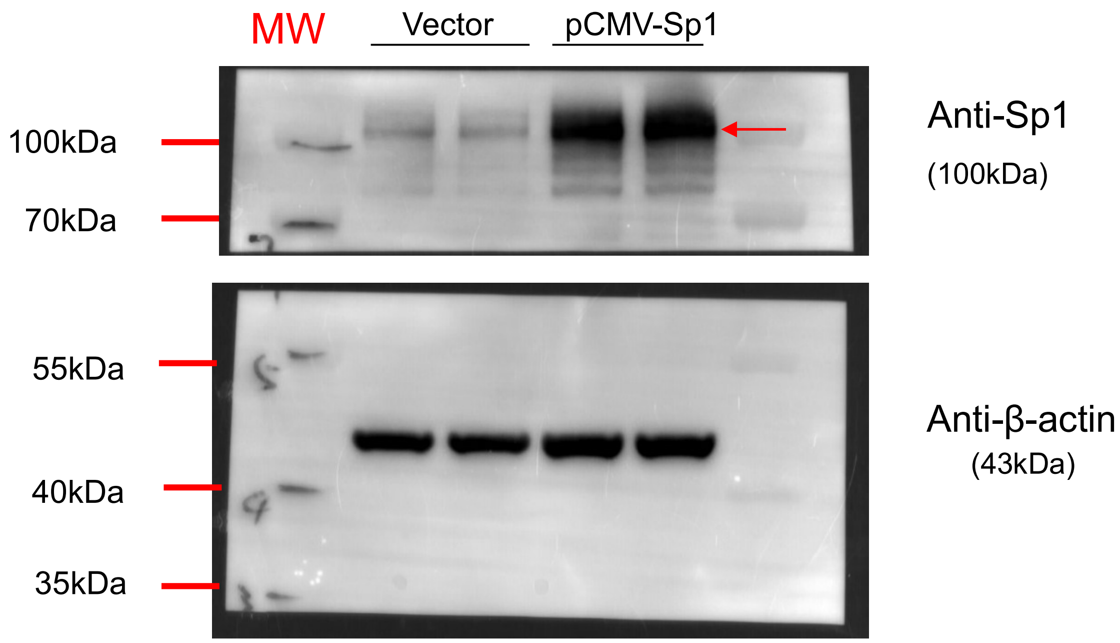

Figure 5G original blot

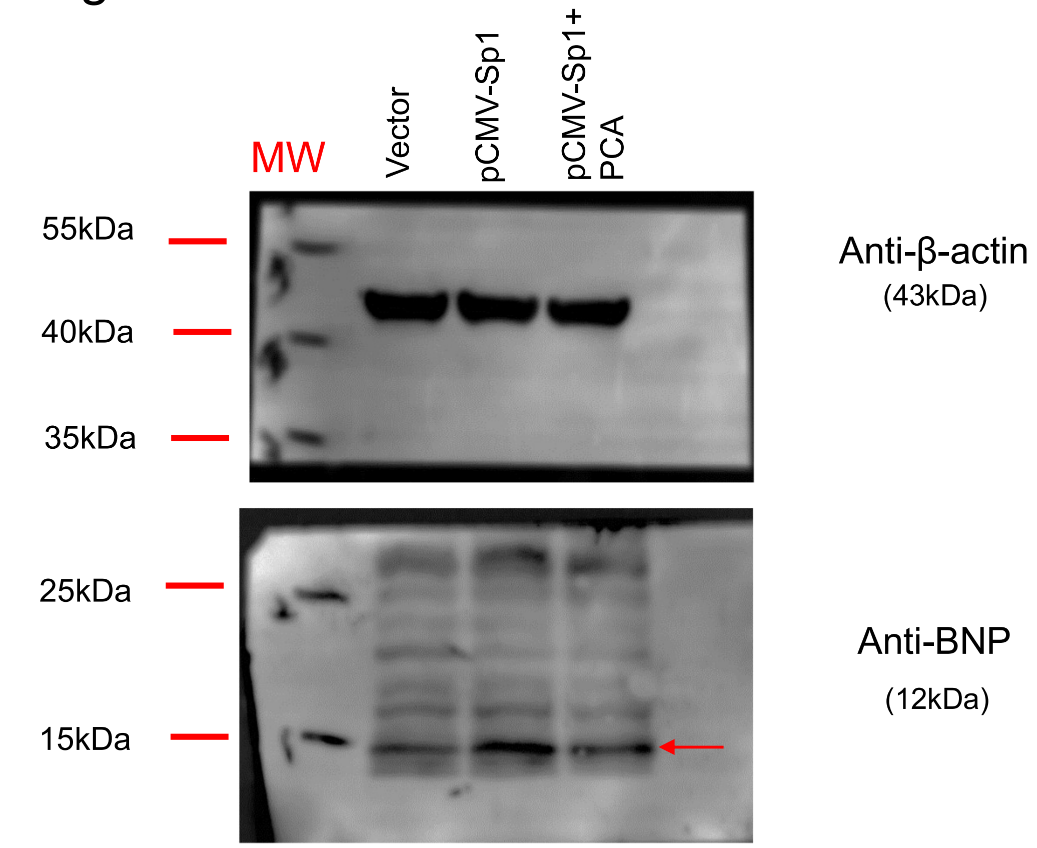

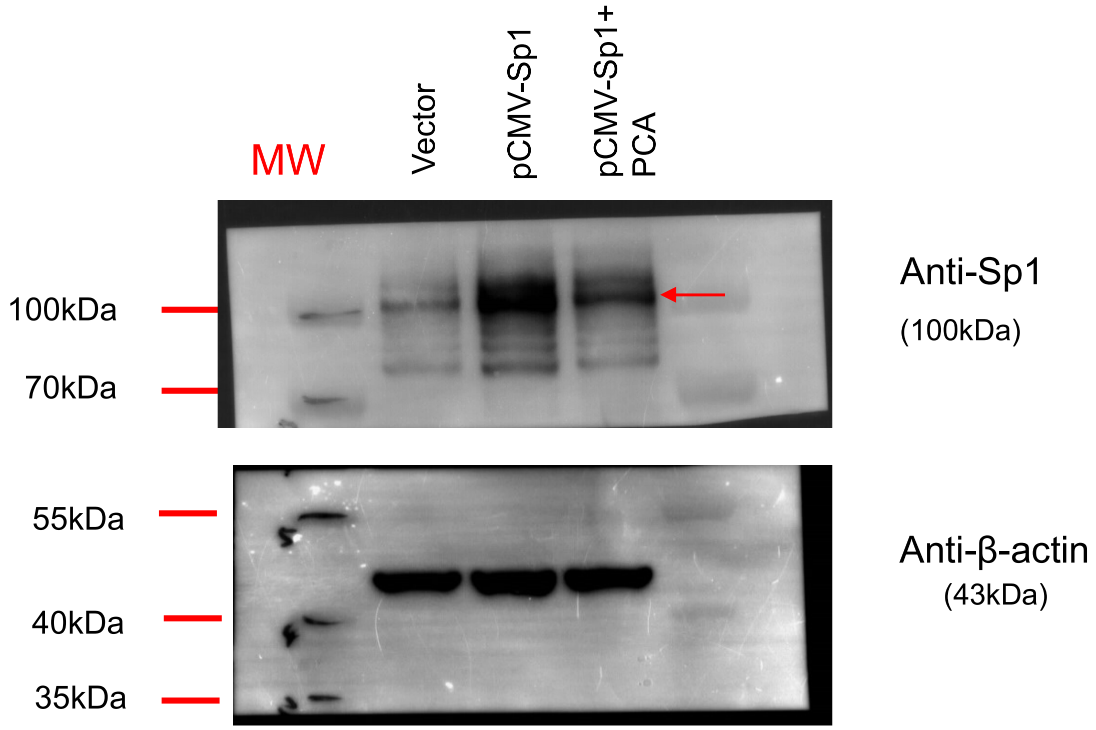

Figure 6C original blot

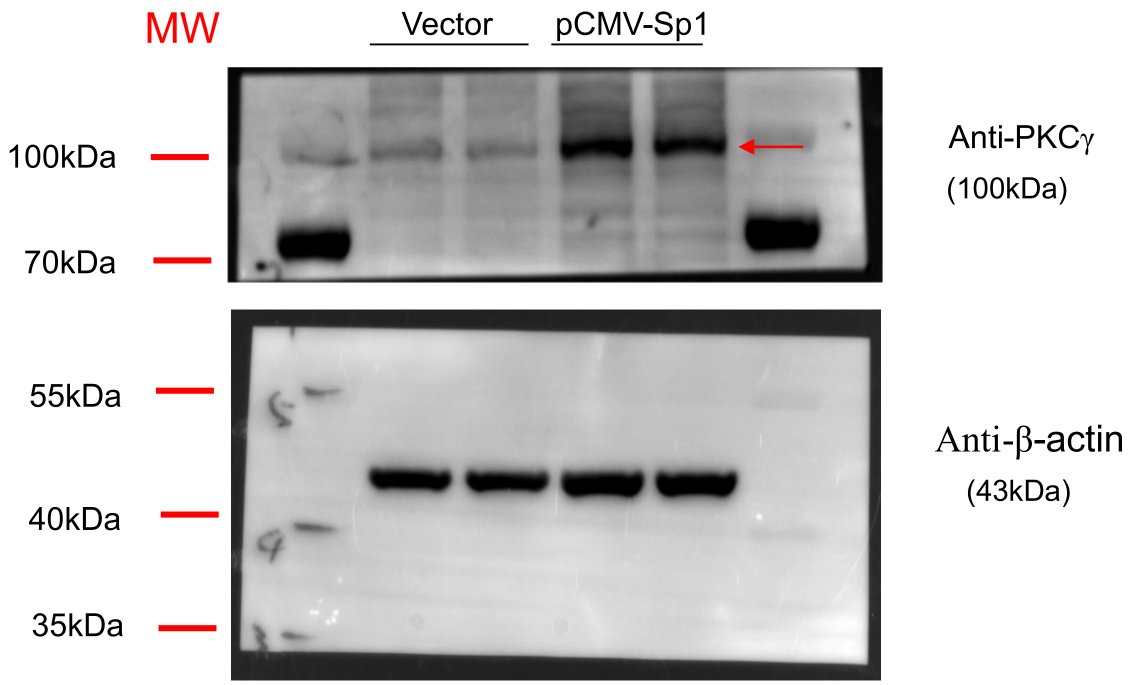

Figure 6F original blot

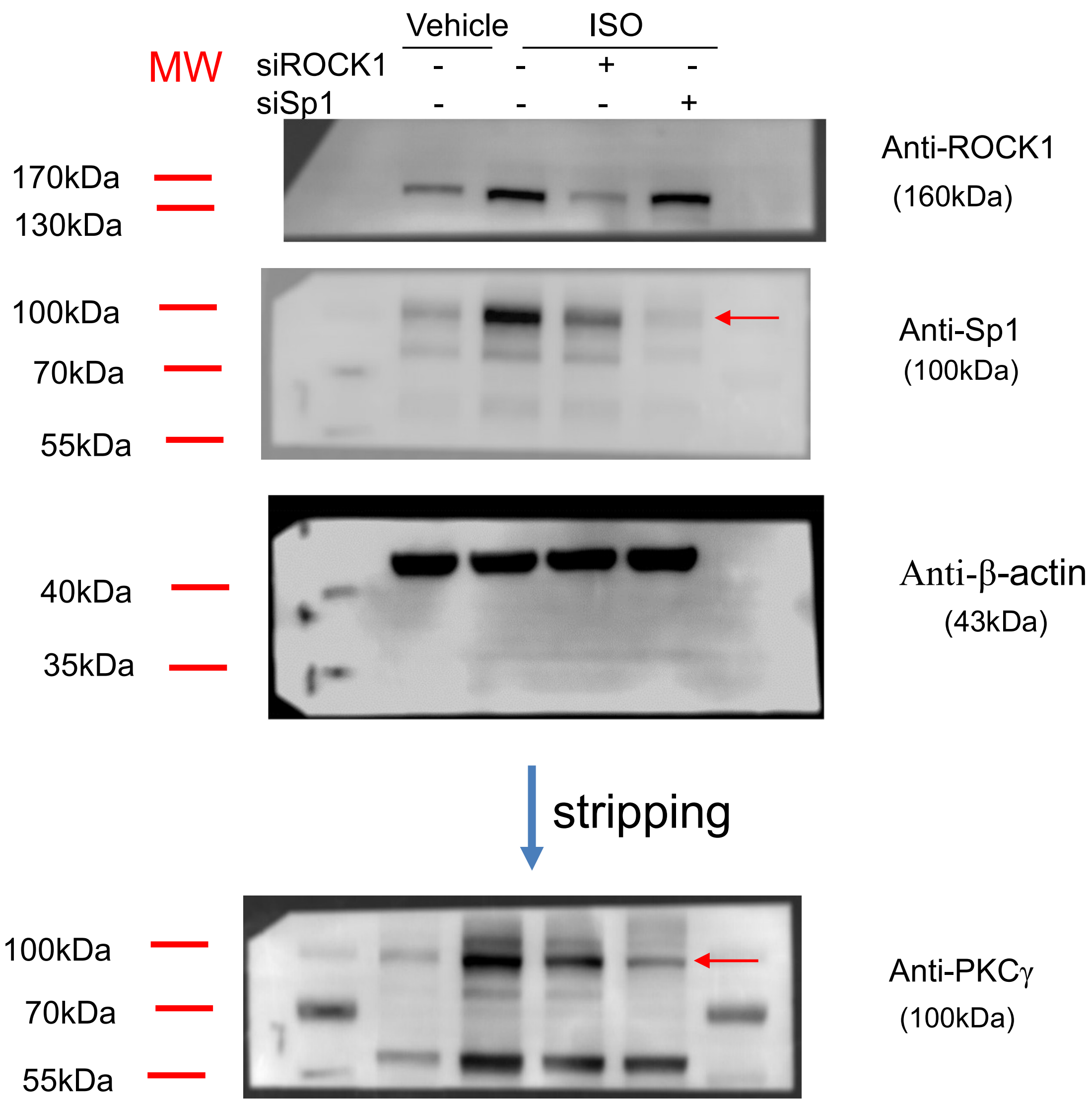

Figure 7A original blot

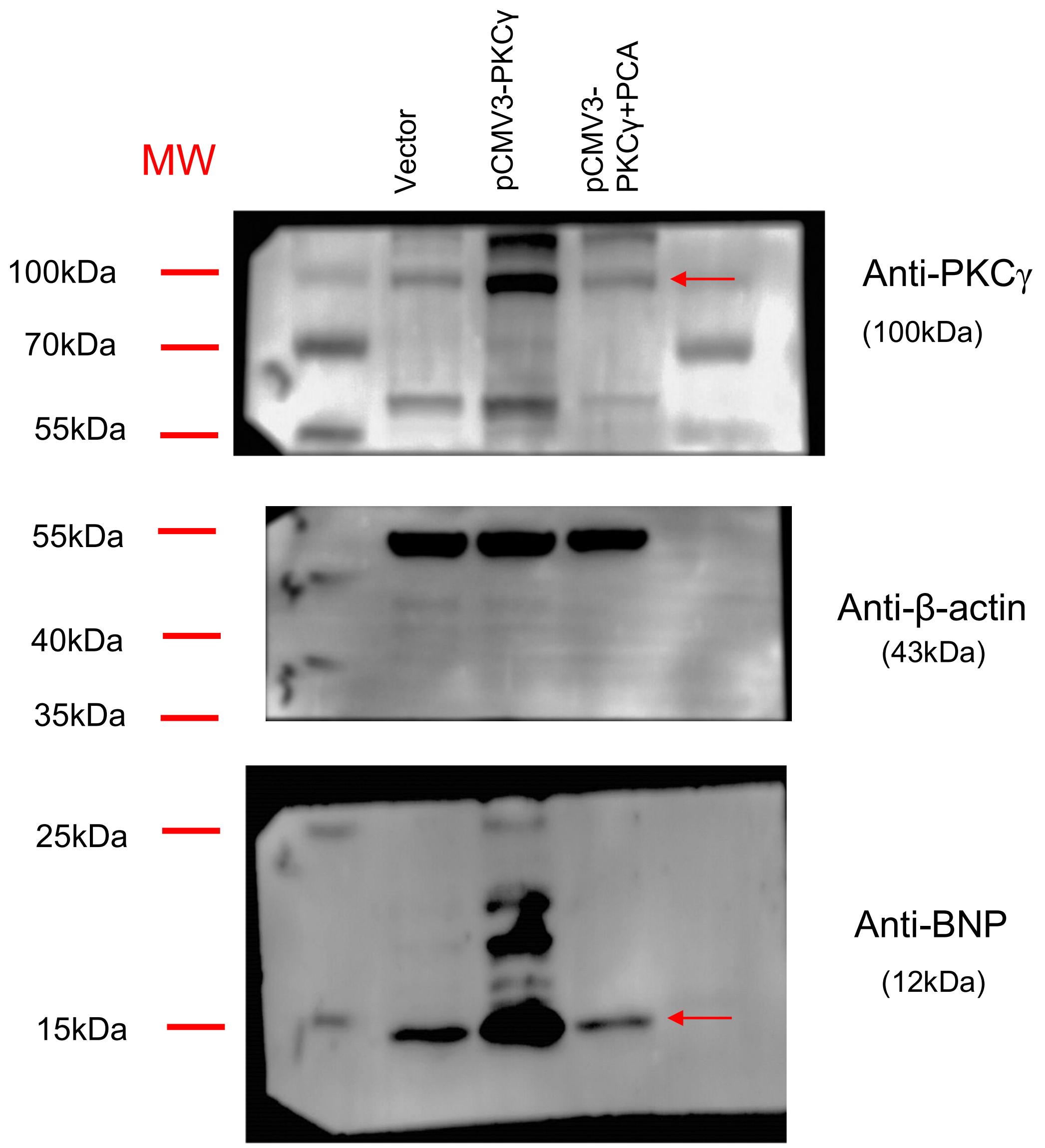

Supplement: Supplementary file 1 — Supplementary Figures. [file 41598_2021_96761_MOESM1_ESM.pdf]
